# Five-year efficacy and safety of guselkumab for moderately to severely active Crohn’s disease: Results from the phase 2 GALAXI 1 trial

**DOI:** 10.1093/ibd/izag055

**Published:** 2026-05-01

**Authors:** Anita Afzali, Silvio Danese, Remo Panaccione, David T Rubin, Bruce E Sands, Walter Reinisch, Julián Panés, Rian Van Rampelbergh, Nat A Terry, Leonardo Salese, Marion L Vetter, Jacqueline Yee, Christopher Corbett, Wilbert van Duijnhoven, Tadakazu Hisamatsu, Jane M Andrews, Geert R D’Haens, the GALAXI 1 investigators, Oleksandr Oliinyk, Leonid Bilianskyi, Jadwiga Gniady-Jastrzebska, Robert Petryka, Tomasz Arlukowicz, Piotr Gietka, Marcin Zmudzinski, Syed Mumtaz, Douglas Wolf, Katarzyna Wojcik, George Duvall, Monika Augustyn, Rafal Filip, Dino Tarabar, Alexander Tkachev, Ursula Seidler, Eran Zittan, Juris Pokrotnieks, Oksana Shchukina, Andro Machavariani, Laura Loy, Niazy Abu-farsakh, Pesegova Marina, Slobodan Sreckovic, Martin Laclav, Shu-Chen Wei, Daniel Suiter, Aleksey Borsuk, Xavier Hebuterne, Carsten Buning, Adi Lahat-Zok, Wit Danilkiewicz, Bernadetta Frysna, Ivana Jovicic, Olena Datsenko, Maninder Guram, Animesh Jain, Zahid Rashid, Sonja Heeren, Natallia Shulga, Ivan Timkin, Srdjan Gornjakovic, Milan Lukas, Romain Altwegg, Ariadne Desjeux, Jean-Marie Reimund, Manana Giorgadze, Christoph Jochum, Hiroaki Ito, Katsuhiko Nakai, Tomohisa Takagi, Osamu Zaha, Changhwan Choi, Taeoh Kim, Jonghun Lee, Ieva Stundiene, Ida Normiha Hilmi, Rosaida Hj Md Said, Jaroslaw Leszczyszyn, Diana Abdulganieva, Yulia Fominykh, Svetlana Maksyashina, Jozef Balaz, Manuel Van Domselaar, Taylan Kav, Patrick Dennis, Patricia Henry, Robert Holmes, Christopher Johnson, Matthew McBride, Harry Sarles, Gregory Moore, Ruslan Yakubtsevich, Vinciane Muls, Stevan Trbojevic, Waqqas Afif, Charles Bernstein, Ivo Klarin, Zuzana Serclova, Miroslava Volfova, Pierre Desreumaux, Cyrielle Gilletta de Saint Joseph, Xavier Roblin, Lucine Vuitton, Kakhaber Chelidze, Tanja Kuehbacher, Ioannis Koutroubakis, Michele Cicala, Walter Fries, Antonio Gasbarrini, Nobuo Aoyama, Yoshito Hayashi, Fumihito Hirai, Norkiyuki Horiki, Namiko Hoshi, Tomoki Inaba, Ishida Hiroyasu, Atsuo Maemoto, Takayuki Matsumoto, Kayoko Matsushima, Satoshi Motoya, Masaki Taruishi, Mohammed Rashid, Jaeyoung Chun, Young-Ho Kim, Dong Il Park, Ala Sharara, Laimas Jonaitis, Gjorgi Deriban, James Brooker, Beata Gawdis-Wojnarska, Barbara Wozniak-Stolarska, Pavel Andreev, Vladimir Simanenkov, Vasiliy Trofimov, Igor Jovanovic, Natasa Zdravkovic, Xavier Aldeguer i Mante, Vicent Hernandez Ramirez, Hale Akpinar, Gurkan Celebi, Hulya Hamzaoglu, Juan Fernandez, Jayaprakash Kamath, Nicole Palekar, Jatinder S Pruthi, David Rausher, Timothy Ritter

**Affiliations:** Division of Gastroenterology and Hepatology, University of Cincinnati College of Medicine, Cincinnati, OH, United States; Gastroenterology and Endoscopy, IRCCS Ospedale San Raffaele and University Vita-Salute San Raffaele, Milan, Italy; Inflammatory Bowel Disease Unit, University of Calgary, Calgary, AB, Canada; Inflammatory Bowel Disease Center, University of Chicago Medicine, Chicago, IL, United States; Dr. Henry D. Janowitz Division of Gastroenterology, Icahn School of Medicine at Mount Sinai, New York, NY, United States; Division of Gastroenterology and Hepatology, Medical University of Vienna, Vienna, Austria; Inflammatory Bowel Diseases Unit, Hospital Clínic de Barcelona, Institut d’Investigacions August Pi i Sunyer, Centro de Investigación Biomédica en Red de Enfermedades Hepáticas y Digestivas, Barcelona, Spain; Johnson & Johnson, Research & Development, Antwerp, Belgium; Johnson & Johnson, Research & Development, Spring House, PA, United States; Johnson & Johnson, Research & Development, Spring House, PA, United States; Johnson & Johnson, Research & Development, Spring House, PA, United States; Johnson & Johnson, Research & Development, Spring House, PA, United States; Johnson & Johnson, Research & Development, High Wycombe, United Kingdom; Johnson & Johnson, Research & Development, Antwerp, Belgium; Department of Gastroenterology and Hepatology, Kyorin University, Tokyo, Japan; IBD Service, Royal Adelaide Hospital and Adelaide University, Adelaide, SA, Australia; Department of Gastroenterology, Amsterdam University Medical Centers, Amsterdam, the Netherlands

**Keywords:** Crohn’s disease, efficacy, guselkumab, long-term follow-up, safety

## Abstract

**Background:**

The randomized, phase 2 GALAXI 1 study evaluated efficacy and safety of guselkumab, a dual-acting IL-23p19 subunit inhibitor, in participants with moderately to severely active Crohn’s disease. Efficacy and safety through 5 years are reported.

**Methods:**

After completing the 48-week, treat-through GALAXI 1 main study, participants could enter the long-term extension (LTE) and continue the subcutaneous maintenance regimen assigned at randomization. Efficacy for the combined guselkumab dose groups was analyzed by (1) as-observed analysis of LTE participants, (2) nonresponder imputation (NRI) analysis of LTE participants, and (3) NRI analysis of primary efficacy analysis set (all [week 0] randomized participants). The study was not designed for statistical comparisons between groups.

**Results:**

Of 185 guselkumab-randomized participants in the primary efficacy analysis set, 151 participated in the LTE. Among those continuing treatment, with available data (as observed), 97.7% (n = 85 of 87) achieved clinical remission, 71.3% (n = 62 of 87) achieved endoscopic response, and 51.7% (n = 45 of 87) achieved endoscopic remission at week 240. Results from NRI analyses also showed durability of efficacy (eg, week-240 clinical remission: 59.2% [n = 84 of 142], LTE analysis set; 46.0% [n = 81 of 176], primary efficacy analysis set). High rates of clinical and endoscopic remission at week 240 were maintained in biologic-naive participants (97.8% [n = 45 of 46] and 54.3% [n = 25 of 46], respectively [as-observed]) and participants with an inadequate response or intolerance to biologic therapy (97.1% [n = 34 of 35] and 54.3% [n = 19 of 35], respectively [as-observed]). Event rates of serious adverse events, discontinuations due to adverse events, and serious infections were low in guselkumab-treated participants.

**Conclusions:**

Long-term guselkumab treatment showed sustained clinical and endoscopic efficacy through week 240. The long-term safety data were consistent with those previously presented in approved indications.

**Clinical trial registration:**

A Study of the Efficacy and Safety of Guselkumab in Participants With Moderately to Severely Active Crohn’s Disease (GALAXI), NCT03466411, https://clinicaltrials.gov/study/NCT03466411

## Graphical Abstract

**Graphical Abstract:**
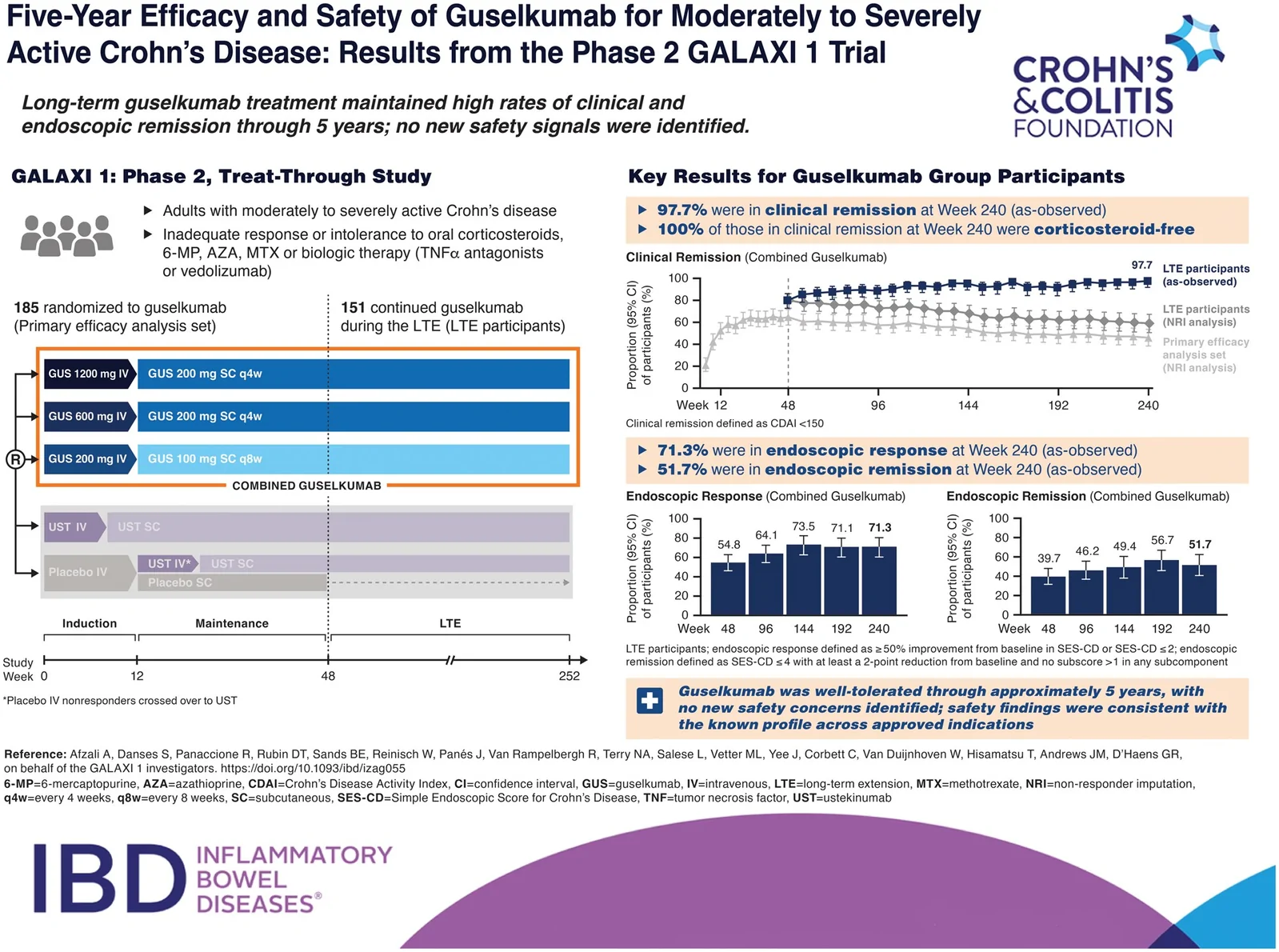

Key Messages *What is already known?*

Through week 48 of the phase 2 GALAXI 1 study, guselkumab (as intravenous induction/subcutaneous maintenance) showed clinical and endoscopic efficacy in participants with moderately to severely active Crohn’s disease; there were no unexpected safety findings.

*What is new here?*

Based on extended follow-up, high rates of clinical and endoscopic remission were sustained through 5 years with continued guselkumab treatment in patients who were biologic-naïve or biologic-exposed; safety was consistent with previous reports.

*How can this study help patient care?*

The findings, which to date represent the longest follow-up for an IL-23p19 subunit inhibitor, highlight guselkumab as a long-term therapy option with durable efficacy and safety in patients with Crohn’s disease, addressing a critical need for durable remission in clinical practice.

## Introduction

Crohn’s disease, a chronic and progressive inflammatory bowel disease with substantial quality of life burden,^1^ requires long-term management.^2^ The disease presentation is heterogeneous, as lesions can occur at sites throughout the gastrointestinal tract; moreover, some patients experience extraintestinal manifestations.^3^ Approximately 80% of patients with Crohn’s disease will require surgery within their lifetime.^3^

Conventional therapies, including corticosteroids, methotrexate, azathioprine, 6-mercaptopurine, and advanced therapies, including biologics and novel oral agents, are used in the treatment of Crohn’s disease; however, some patients lose treatment response over time and/or experience intolerance. Despite approval of multiple advanced therapies, few studies have reported durable 5-year outcomes in Crohn’s disease, particularly for therapies in the interleukin (IL)-23 inhibitor class. Long-term endoscopic data are of particular interest, as endoscopy is considered a strong predictor for long-term outcomes,^4^ and endoscopic remission in particular has been associated with reduced disease progression and fewer hospitalizations and surgeries.^5^

Guselkumab is a selective, dual-acting IL-23p19 subunit inhibitor that potently blocks IL-23 and binds to CD64, a receptor on immune cells that produce IL-23.^6^ Guselkumab is approved for the treatment of moderately to severely active Crohn’s disease and ulcerative colitis in adults.^7^^,^^8^ In other indications (psoriatic diseases), guselkumab has demonstrated long-term efficacy and safety through at least 5 years.^9^^,^^10^

The GALAXI clinical development program is composed of 3 randomized, double-blind, placebo-controlled and active comparator (ustekinumab)–controlled treat-through studies of guselkumab in participants with moderately to severely active Crohn’s disease and documented inadequate response or intolerance to prior conventional or biologic therapy.^11–13^ GALAXI 1 is a 48-week, randomized, double-blind, parallel-group phase 2 dose-finding study,^11^^,^^12^ while GALAXI 2 and GALAXI 3 are identical 48-week phase 3 confirmatory studies.^13^ In all 3 studies, participants treated with guselkumab intravenous (IV) induction and subcutaneous (SC) maintenance regimens achieved high rates of clinical and endoscopic endpoints through week 48.^11–13^ Guselkumab was well tolerated, and the safety profile was similar to observations from other approved indications.^7^^,^^8^

GALAXI participants were able to enter the open-label long-term extension (LTE) to receive up to approximately 4 additional years of treatment. We report efficacy and safety results through the end of the phase 2 GALAXI 1 LTE, representing a total of approximately 5 years of guselkumab treatment.

## Methods

### Study design and participants

**Figure 1:**
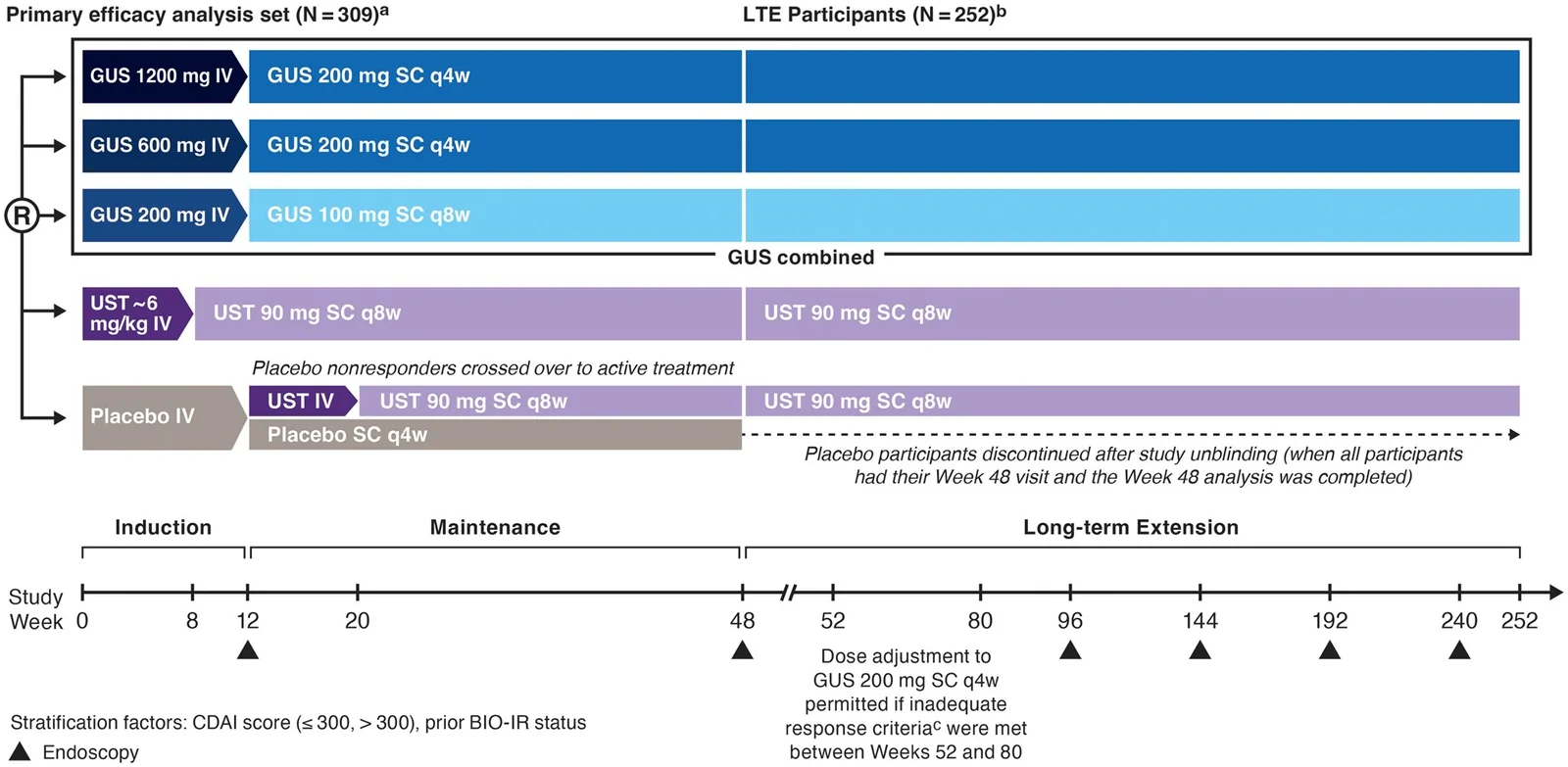
Overview of GALAXI 1 study design. ^a^Randomized participants who received at least 1 dose of study intervention (including a partial dose). ^b^Randomized participants who entered the long-term extension (LTE) phase of the study and received at least 1 dose of study intervention (including a partial dose) during the LTE phase. ^c^Inadequate response is defined as not being in clinical response and having a CDAI score of ≥220 points. Clinical response is defined as a reduction from baseline (ie, week 0) in the CDAI score of ≥100 points or being in clinical remission (Crohn’s Disease Activity Index [CDAI] <150). Note: the last dose of study drug was administered at week 232 (for every 8 weeks [q8w] dosing) or week 236 (for every 4 weeks [q4w] dosing). BIO-IR, history of inadequate response or intolerance to biologic therapy; GUS, guselkumab; IV, intravenous; PBO, placebo; R, randomization; SC, subcutaneous; UST, ustekinumab.

Detailed descriptions of the treat-through study design and inclusion criteria for GALAXI 1 (NCT03466411; study dates: May 10, 2018 through June 25, 2024) have been reported.^11^^,^^12^ Features relevant to the LTE are summarized subsequently and shown in Figure 1.

At baseline, adults with moderately to severely active Crohn’s disease with an inadequate response or intolerance to conventional therapy (ie, oral corticosteroids, 6-mercaptopurine, azathioprine, or methotrexate) or biologic therapy (ie, tumor necrosis factor [TNF] antagonists or vedolizumab) were randomized in a 1:1:1:1:1 ratio to guselkumab 1200 mg IV → 200 mg SC every 4 weeks [q4w]; guselkumab 600 mg IV → 200 mg SC q4w; guselkumab 200 mg IV → 100 mg SC every 8 weeks [q8w]; ustekinumab (∼6 mg/kg IV at week 0, then 90 mg SC q8w; reference arm); or placebo. Placebo-randomized participants who were not in clinical response after induction (week 12) crossed over to receive ustekinumab rescue therapy.

At week 48, participants who, based on the investigator’s assessment, were improving with guselkumab treatment (based on week-48 clinical and endoscopic assessments) entered the LTE to receive approximately 4 additional years of treatment. LTE participants continued to receive the SC treatment regimen that they received at their last study visit prior to the start of the LTE, which was the same treatment they were randomized to at week 0 (except for participants randomized to placebo who met inadequate response criteria and crossed over to ustekinumab). Participants meeting inadequate response criteria (defined as not being in clinical response AND a Crohn’s Disease Activity Index [CDAI] score ≥220) between weeks 52 and 80 were eligible for a dose adjustment. Participants receiving placebo, ustekinumab, or guselkumab 100 mg SC q8w at LTE entry had their treatment adjusted to the guselkumab 200 mg SC q4w dose. Those already receiving guselkumab 200 mg SC q4w at LTE entry received a sham treatment adjustment (only while the study was still blinded).

Participants continued to receive blinded study drug during the LTE until all week-48 study visits were completed and the study was unblinded to the investigative sites (ie, after the GALAXI 1 week 48 database lock and completion of the week 48 analysis). Additionally, participants in the placebo, guselkumab 100 mg SC q8w, and ustekinumab groups who met criteria for dose adjustment between weeks 52 and 80 transitioned to guselkumab 200 mg SC q4w. After unblinding, placebo participants who did not meet the inadequate response criteria prior to unblinding (and thus were ineligible for dose adjustment) were discontinued from the study. At the time of unblinding, participants on active treatment (guselkumab or ustekinumab) continued their treatment open label for the remainder of the LTE. Participants still in the study between weeks 52 and 80 after unblinding were eligible for dose escalation if inadequate response criteria were met.

The GALAXI 1 study complied with ethical guidelines, including Declaration of Helsinki, Good Clinical Practice guidelines, and applicable local regulations. An institutional review board or ethics committee at each participating site approved the protocol. All participants provided written informed consent.

### Study drug administration

Administration of IV induction and SC maintenance regimens has been described previously.^11^^,^^12^ Information about concomitant Crohn’s disease medications is provided in the Supplementary Appendix.

### Assessments

The CDAI score was assessed q4w through week 48 and at least q8w from weeks 56 through 240. Video ileocolonoscopic examinations were performed at screening and weeks 12, 48, 96, 144, 192, and 240. We evaluated endoscopic endpoints using the Simple Endoscopic Score for Crohn’s Disease (SES-CD), scored by central readers blinded to the treatment assignment and study visit.

Histologic assessments were performed using biopsy samples collected during ileocolonoscopy from each of 3 predefined anatomic locations: terminal ileum, splenic flexure, and rectum (as clinically feasible). Biopsies were collected from representative areas that were consistent with the inflammation status visually observed during endoscopy. Histologic disease activity was scored by central readers blinded to the treatment assignment, site, and study visit and evaluated using the Robarts Histopathology Index criteria.

Inflammatory biomarkers (C-reactive protein [CRP] and fecal calprotectin) were assessed from blood and stool samples collected every 16 weeks (q16w) (q8w for CRP from weeks 48 to 96) during the LTE.

Participants completed the Inflammatory Bowel Disease Questionnaire (IBDQ)^14^ at weeks 0, 8, 12, 24, and 48 and q16w during the LTE.

At each study visit, participants were evaluated for adverse events (AEs) and serious adverse events (SAEs). The following definition was used for SAEs: life-threatening AEs, AEs resulting in death, AEs requiring new or prolonged hospitalization, AEs resulting in persistent/significant disability or incapacity, birth defects or congenital anomalies, suspected transmission of infectious agent via a medicinal product, or other medically important AEs. Clinical safety laboratory assessments, including blood chemistry, liver function tests, and hematology were performed q16w during the LTE.

Blood samples were collected for predose serum guselkumab concentration measurements q8w from week 48 to week 96, then q16w. Serum antidrug antibodies (ADAs) were measured q16w. The assays used for pharmacokinetic and immunogenicity assessments have been described previously.^11^

Medical resource utilization was evaluated throughout the study (weeks 0 to 240), including Crohn’s disease–related surgeries, emergency room visits, hospitalizations, and procedures.

### Outcomes

GALAXI 1 primary and major secondary endpoints have been previously reported.^12^ All endpoints assessed during the LTE were exploratory. Definitions for efficacy endpoints evaluated during the LTE are summarized in Table S1, including clinical remission, durable clinical remission, corticosteroid-free clinical remission, 2-item patient-reported outcome (PRO-2) remission, endoscopic response, endoscopic remission, histologic response (per Robarts Histopathology Index criteria), and inflammatory biomarker (CRP and fecal calprotectin) normalization. Participant-level composite endpoints included (1) clinical remission *and* endoscopic response, (2) deep remission (clinical remission *and* endoscopic remission), and (3) histoendoscopic response (histologic response *and* endoscopic response).

Subgroup analyses of clinical and endoscopic efficacy outcomes were conducted in (1) participants with a prior inadequate response or intolerance to ≥1 biologic therapies at an approved dose for Crohn’s disease (BIO-IR) and (2) participants who had never been exposed to biologic therapy (BIO-naive).

Safety endpoints included treatment-emergent AEs, SAEs, deaths, infections, serious infections, and laboratory assessment changes through end of study. Protocol-specified events of special/clinical interest included cases of malignancies, active tuberculosis, opportunistic infections, venous thromboembolism (VTE), major adverse cardiovascular events (MACE), clinically important hepatic disorders (defined as hepatic disorder SAEs or AEs leading to discontinuation), anaphylactic reactions, and serum sickness reactions.

Serum trough guselkumab concentrations over time were summarized by dose group. Immunogenicity outcomes included the incidence of antibodies to guselkumab or ustekinumab and the incidence of neutralizing antibodies.

### Statistical analysis

Long-term data were summarized descriptively for the combined guselkumab group (ie, 200 mg IV q4w → 100 mg SC q8w, 600 mg IV → 200 mg SC q4w, and 1200 mg IV q4w → 200 mg SC q4w dose groups) due to the relatively small numbers of participants in each randomized dose group. Key efficacy and safety data for the ustekinumab reference arm are summarized in the Supplementary Appendix. No formal comparisons between treatment groups, including ustekinumab, were included in the study design.

Efficacy was analyzed using 3 approaches: (1) LTE efficacy analysis set (ie, randomized participants who entered the LTE), using an as-observed approach; (2) LTE efficacy analysis set, using a nonresponder imputation (NRI) approach; and (3) primary efficacy analysis set (ie, including all randomized participants), using an NRI approach. Details regarding the populations and analysis approaches are summarized in Table S2.

Twelve participants completed the study at week 144 before implementation of a protocol amendment that extended the LTE through 240 weeks. These participants were excluded from the denominator for clinical endpoint analyses at week 152 and after and from the denominator for endoscopic endpoint analyses at week 144 and thereafter.

Safety data were summarized during the entire study period for GALAXI 1 (ie, from week 0 to end of the LTE) for the full safety analysis set of all randomized participants who received ≥1 study treatment dose (full or partial). AEs, SAEs, and AEs leading to study drug discontinuation were summarized by MedDRA (Medical Dictionary for Regulatory Activities) (version 27.0) preferred term and reported as proportion of participants and exposure-adjusted event rates (number of events per 100 patient-years [PY]).

ADAs to guselkumab and ustekinumab were evaluated in participants who had received ≥1 dose of guselkumab or ustekinumab, respectively, and had samples appropriate for detection of antibodies.

There was no specific sample size determination for the LTE study. Sample size and power considerations were applicable for the main study only and have been described previously.^11^^,^^12^

## Results

### Participants

**Figure 2:**
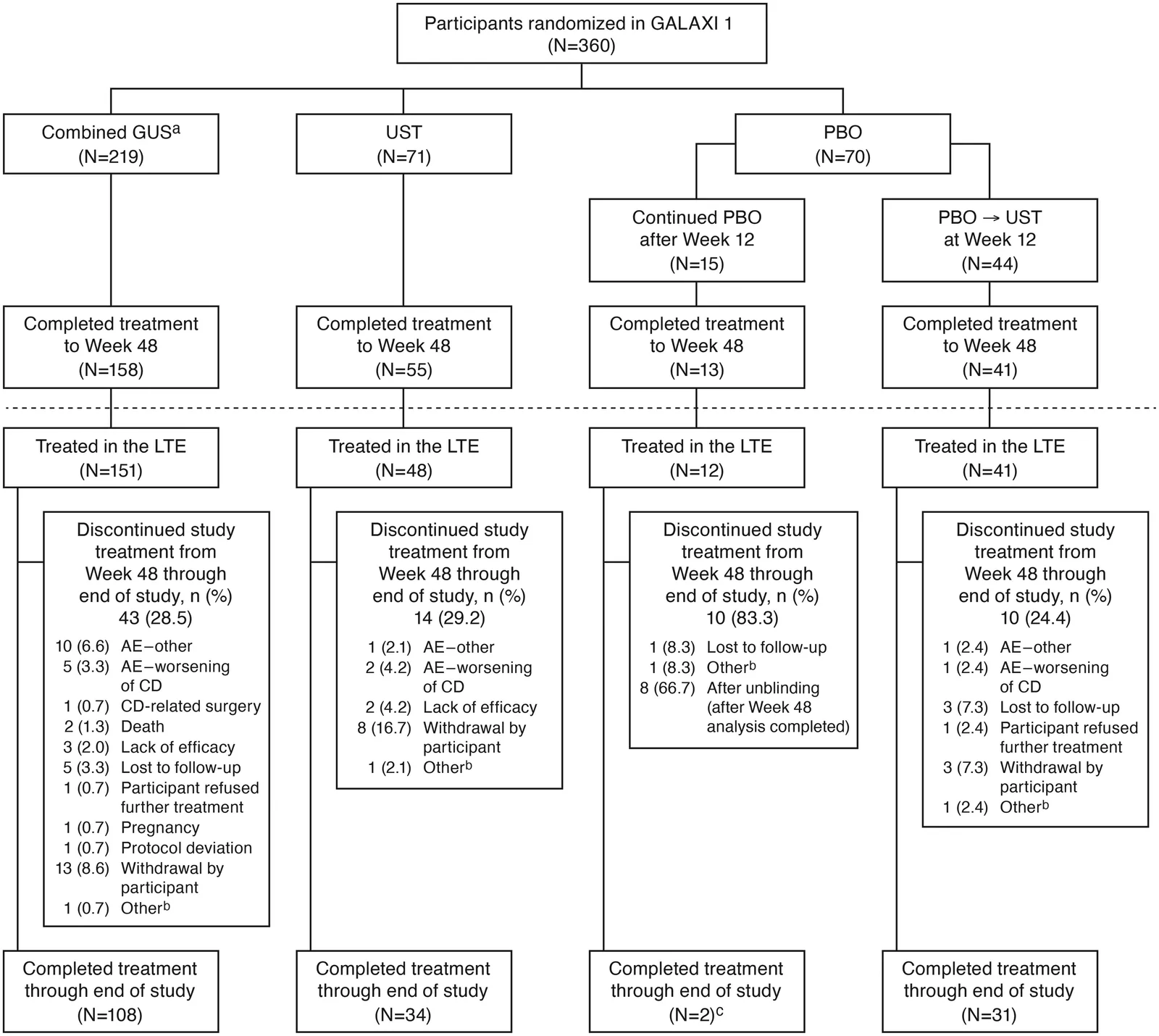
Treatment disposition from week 48 through end of study. ^a^Includes participants receiving either the guselkumab (GUS) 100 mg subcutaneous (SC) every 8 weeks (q8w) or 200 mg SC every 4 weeks (q4w) maintenance dose. ^b^Other reasons included desire for childbearing, participant decision related to pregnancy, participant moved to another country, and slow healing from back surgery with lack of communication. ^c^Both participants underwent dose adjustment to GUS 200 mg SC q4w prior to unblinding; 1 participant completed treatment but discontinued the study; 1 completed the study. AE, adverse event; CD, Crohn’s disease; LTE, long-term extension; PBO, placebo; UST, ustekinumab.

Of 309 primary efficacy analysis set participants who underwent randomization at baseline (week 0) and received treatment in GALAXI 1, 252 entered the LTE, including 151 participants in the combined guselkumab dose group, 89 ustekinumab-treated participants (n = 48 randomized to ustekinumab; n = 41 placebo → ustekinumab week 12 crossover), and 12 placebo participants (Figure 2). A total of 175 (69.4%) participants completed treatment through end of the study, including 108 (71.5%) guselkumab-treated participants, 65 (73.0%) ustekinumab-treated participants (n = 34 ustekinumab-randomized; n = 31 placebo → ustekinumab), and 2 placebo group participants (both of whom underwent a dose adjustment to guselkumab 200 mg q4w SC prior to unblinding).

From week 48 to the end of study, 43 (28.5%) of 151 participants in the combined guselkumab group and 14 (29.2%) of 48 ustekinumab-randomized participants discontinued study treatment, most commonly due to withdrawal by participant, AEs other than worsening of Crohn’s disease, loss to follow-up, or AEs of worsening Crohn’s disease (Figure 2).

Baseline (week 0) demographic and disease characteristics for LTE participants in the combined guselkumab group are summarized in Table 1. Approximately half (51.7% [n = 78 of 151]) were BIO-IR, while 42.4% (n = 64 of 151) were BIO-naive. Most BIO-IR participants (n = 67 of 78) had an inadequate response or intolerance to an anti-TNF therapy only, while 8 of 78 had an inadequate response or intolerance to both anti-TNF and vedolizumab (vedolizumab only: 3 of 78). Overall, baseline disease characteristics were generally similar among the BIO-IR and BIO-naive participants in the guselkumab group except for Crohn’s disease duration, which was longer in the BIO-IR subgroup (median 9.4 years [interquartile range: 6.1-17.1 years] vs 2.5 years [interquartile range: 1.2-5.7 years], respectively) (data not shown). Baseline characteristics for the ustekinumab reference group, summarized in Table S3, were similar to the combined guselkumab group.

**Table 1:** Demographics and disease characteristics at GALAXI 1 baseline (week 0) for the combined guselkumab group (LTE participants) **(n = 151).**

**Age, y**	
**Mean ± SD**	39.1 ± 13.8
**Median (IQR)**	36.0 (28.0-50.0)
**Sex**	
**Male**	92 (60.9)
**Female**	59 (39.1)
**Race**	
**White**	127 (84.1)
**Asian**	15 (9.9)
**American Indian or Alaska Native**	2 (1.3)
**Black or African American**	1 (0.7)
**Native Hawaiian or other Pacific Islander**	1 (0.7)
**Not reported**	5 (3.3)
**Ethnicity**	
**Not Hispanic or Latino**	138 (91.4)
**Hispanic or Latino**	7 (4.6)
**Not reported**	6 (4.0)
**Weight, kg**	70.8 ± 16.2
**CD duration, y**	
**Mean ± SD**	9.3 ± 10.2
**Median (IQR)**	6.7 (2.2-11.6)
**CDAI score**	
**Mean ± SD**	301.4 ± 55.1
**Median (IQR)**	294.0 (253.0-341.0)
**PRO-2 score**	143.3 ± 41.5
**Stool frequency >3**	119 (78.8)
**Abdominal pain >1**	141 (93.4)
**SES-CD**	
**Mean ± SD**	12.8 ± 7.5
**Median (IQR)**	11.0 (7.0-18.0)
**CRP concentration, mg/L**	
**Mean ± SD**	15.1 ± 24.7
**Median (IQR)**	5.6 (1.6-17.6)
**Fecal calprotectin, µg/g**	
**Mean ± SD**	1423.2 ± 2773.5^a^
**Median (IQR)**	596.0 (202.0-1662.0)^a^
**IBDQ score**	126.5 ± 35.2
**Endoscopic disease severity per SES-CD**	
**Moderate (7-16)**	70 (46.4)
**Severe (>16)**	45 (29.8)
**Involved GI areas by central reader**	
**Ileum only**	43 (28.5)
**Colon only**	60 (39.7)
**Ileum and colon**	48 (31.8)
**≥1 open or draining fistula**	19 (12.6)
**Prior and concomitant CD medications**	
**≥1 CD medications taken at baseline**	116 (76.8)
**Immunomodulatory drugs**	52 (34.4)
**Corticosteroids, including budesonide and beclomethasone dipropionate**	53 (35.1)
**Corticosteroids, excluding budesonide and beclomethasone dipropionate**	33 (21.9)
**Prednisone-equivalent dose, mg/d^b^**	22.5 ± 11.4
**Prior inadequate response or intolerance to biologics**	78 (51.7)
**≥1 anti-TNF^c^**	75 (49.7)
**≥2 anti-TNF**	30 (19.9)
**Anti-TNF only**	67 (44.4)
**Vedolizumab**	11 (7.3)
**Vedolizumab and ≥1 anti-TNF**	8 (5.3)
**Biologic-naive**	64 (42.4)
**Biologic experienced without documented inadequate response or intolerance**	9 (6.0)

Values are n (%) or mean ± SD, unless otherwise indicated.

The combined guselkumab group includes all LTE participants receiving either guselkumab 100 mg SC every 8 weeks or guselkumab 200 mg SC every 4 weeks at start of the LTE.

Abbreviations: CD, Crohn’s disease; CDAI, Crohn’s Disease Activity Index; CRP, C-reactive protein; GI, gastrointestinal; IBDQ, Inflammatory Bowel Disease Questionnaire; IQR, interquartile range; LTE, long-term extension; PRO-2, 2-item Patient-Reported Outcome; SC, subcutaneous; SD, standard deviation; SES-CD, Simple Endoscopic Score for Crohn’s Disease; TNF, tumor necrosis factor.

a n = 149.

b Excluding budesonide and beclomethasone dipropionate.

c Anti-TNF includes adalimumab (n = 50), infliximab (n = 53), and certolizumab pegol (n = 4).

At week 48 (ie, start of the LTE), the median CDAI score was 76.0 (mean 87.9), median SES-CD was 4.0 (mean 5.2), median fecal calprotectin level was 121.5 µg/g (mean 527.9 µg/g), and median CRP concentration was 1.9 mg/L (mean 4.9 mg/L) among LTE participants in the combined guselkumab group (Table S4). Few LTE participants (6.0% [n = 9 of 151] in the combined guselkumab group) were receiving corticosteroids (including budesonide and beclomethasone dipropionate) at week 48.

### Efficacy

Efficacy data from the as-observed analysis in LTE participants are summarized subsequently. Corresponding data from the LTE NRI analysis and the primary efficacy analysis set for each outcome are also provided in the figures and/or Supplementary Appendix.

**Figure 3:**
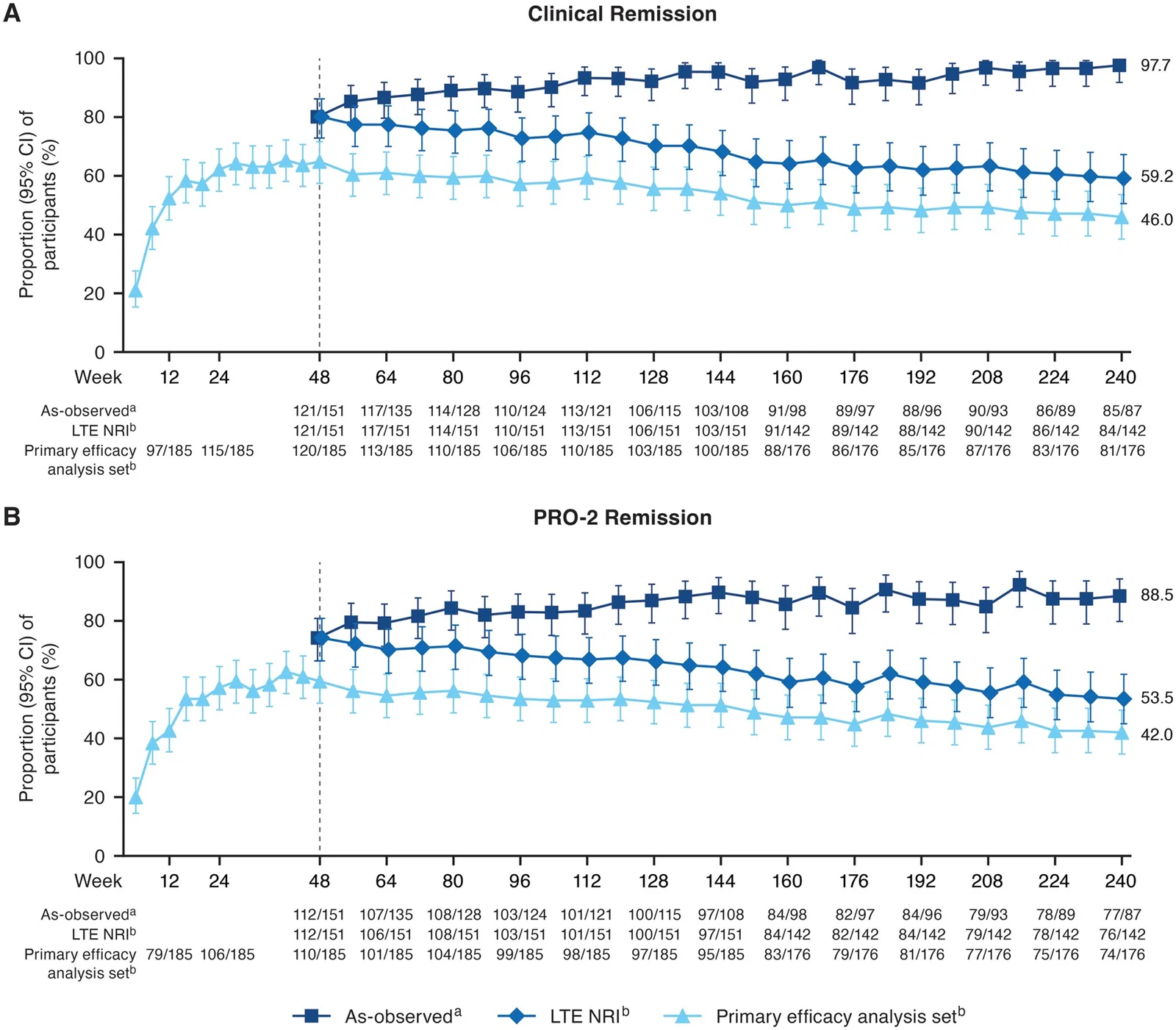
(A) Clinical remission and (B) 2-item patient-reported outcome (PRO-2) remission in the combined guselkumab group by visit through week 240. ^a^Observed rates were evaluated in participants who entered the long-term extension (LTE) and remained on treatment at that specific time point. Participants who dose-adjusted were excluded after the time of dose adjustment. No treatment failure rules were applied. Participants who did not have data at a visit were excluded from analysis for that visit. ^b^Participants with missing data at a particular time point after accounting for intercurrent event strategies were considered not to have achieved the endpoint at that time point. Participants who completed the study at week 144 under an earlier version of the protocol are excluded from the denominator for all analyses thereafter. Clinical remission is defined as Crohn’s Disease Activity Index (CDAI) score <150. PRO-2 remission is defined as abdominal pain (AP) mean daily score ≤1 and stool frequency (SF) mean daily score ≤3, and no worsening of AP or SF from baseline. CI, confidence interval; NRI, nonresponder imputation.

Of LTE participants in the combined guselkumab group who received continuous treatment and had available data, most achieved clinical remission throughout the LTE period (Figure 3A). At week 48, 80.1% (n = 121 of 151) and at week 240, 97.7% (n = 85 of 87) of participants achieved clinical remission (as-observed analysis). Most (90.8% [n = 79 of 87]) LTE participants in clinical remission at week 240 were also in durable clinical remission (Figure S1A). The proportion of participants in corticosteroid-free (90 days) clinical remission was similar to the proportion in clinical remission throughout the LTE (Figure S1B), and all guselkumab participants who were in clinical remission at week 240 were corticosteroid-free.

Most participants receiving corticosteroids at the start of induction were tapered between week 0 and week 48 and remained corticosteroid-free during the LTE (ie, n = 41 of 53 [77.4%]). Few participants in the combined guselkumab group (9.3% [n = 14 of 151]) initiated or increased corticosteroid use during the LTE. Among guselkumab group participants in the LTE receiving corticosteroids at baseline, the median daily prednisone-equivalent corticosteroid dose was 20.0 mg/d (interquartile range: 20-25 mg/d) at week 0 and 0.0 mg/d (interquartile range: 0-0 mg/d) at both week 48 and week 240 (Table S5).

High rates of PRO-2 remission were observed throughout the LTE period in guselkumab-treated participants, with 74.2% (n = 112 of 151) and 88.5% (n = 77 of 87) achieving PRO-2 remission at weeks 48 and 240, respectively (as-observed analysis) (Figure 3B).

Clinical remission rates and PRO-2 remission rates for participants in the primary efficacy analysis set and for the LTE NRI analysis are shown in Figure 3. Overall, the rates of clinical remission or PRO-2 remission increased over time during the main study period, and participants continued to achieve high rates of these outcomes during the LTE.

**Figure 4:**
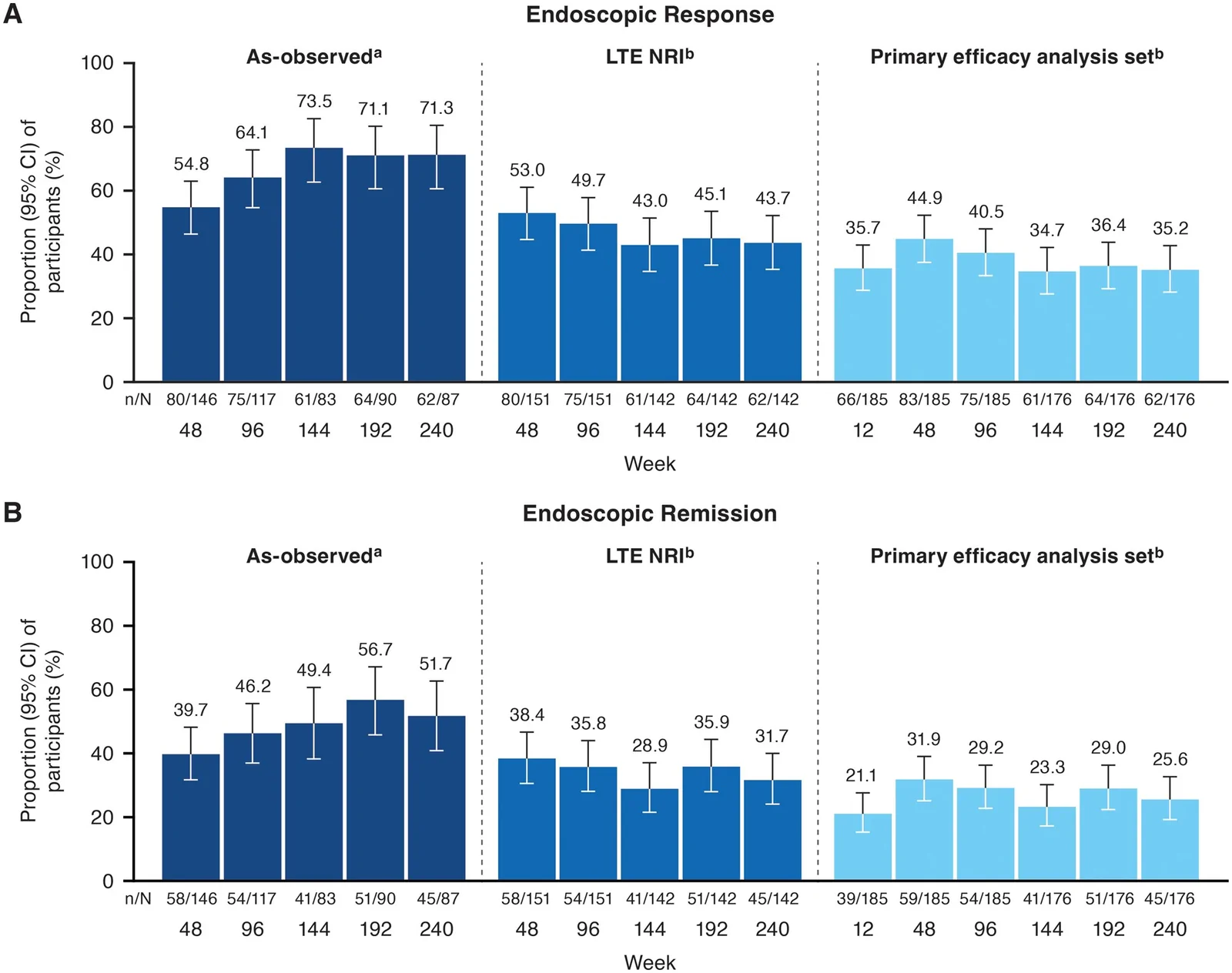
(A) Endoscopic response and (B) endoscopic remission in the combined guselkumab group by visit through week 240. ^a^Observed rates were evaluated in participants who entered the long-term extension (LTE) and remained on treatment at that specific time point. Participants who dose-adjusted were excluded after the time of dose adjustment. No treatment failure rules were applied. Participants who did not have data at a visit were excluded from analysis for that visit. ^b^Participants with missing data at a particular time point after accounting for intercurrent event strategies were considered not to have achieved the endpoint at that time point. Participants who completed the study at week 144 under an earlier version of the protocol were not scheduled to have an ileocolonoscopy from week 144 and were excluded from analysis at week 144 and thereafter. Endoscopic response is defined as ≥50% improvement from baseline in Simple Endoscopic Score for Crohn’s Disease (SES-CD) or SES-CD ≤2. Endoscopic remission is defined as SES-CD ≤4 with at least a 2-point reduction from baseline and no subscore >1 in any subcomponent. CI, confidence interval; NRI, nonresponder imputation.

The proportions of participants in the combined guselkumab group achieving endoscopic response or endoscopic remission were generally sustained throughout the LTE (Figure 4). In the as-observed analysis, 54.8% (n = 80 of 146) achieved endoscopic response at week 48 and 71.3% (n = 62 of 87) at week 240 (Figure 4A). Corresponding rates of endoscopic remission were 39.7% (n = 58 of 146) and 51.7% (n = 45 of 87), respectively (as-observed analysis) (Figure 4B). Results for the primary efficacy analysis set and LTE NRI analysis are also shown in Figure 4.

The proportions of participants in the combined guselkumab group achieving both clinical remission *and* endoscopic response or deep remission (clinical remission *and* endoscopic remission) were generally sustained during the LTE (Figure S2). At week 240, 69.4% (n = 59 of 85) of participants in the combined guselkumab group achieved clinical remission *and* endoscopic response; 38.8% (n = 33 of 85) achieved deep remission (clinical remission *and* endoscopic remission) (as-observed analysis).

IBDQ remission rates (Figure S3) and rates of achieving histologic response (for participants with histologic disease activity at baseline) and histoendoscopic response (Figure S4) were generally sustained during the LTE study among guselkumab-treated participants. The proportions of guselkumab-treated participants with normalization of CRP and normalization of fecal calprotectin were also generally stable throughout the LTE (Figure S5).

Clinical and endoscopic results in subgroups of participants from the combined guselkumab group who were BIO-IR or BIO-naive are shown in Figure S6. At week 240, most participants in both the BIO-IR (97.1% [n = 34 of 35]) and BIO-naive (97.8% [n = 45 of 46]) subgroups were in clinical remission (as-observed analysis) (Figure S6A). For endoscopic outcomes at week 240, 82.9% (n = 29 of 35) of BIO-IR and 63.0% (n = 29 of 46) of BIO-naive participants achieved endoscopic response, while 54.3% (n = 19 of 35) and 54.3% (n = 25 of 46), respectively, achieved endoscopic remission (as-observed analysis) (Figure S6B, C). Corresponding results from the primary efficacy analysis set and LTE NRI analysis are also shown in Figure S6. Interpretation of the subgroup data is limited by small sample sizes.

Clinical and endoscopic efficacy data for participants in the ustekinumab reference arm are summarized in Figure S7.

### Dose adjustment

A total of 35 participants met the criteria for dose adjustment between weeks 52 and 80, including 26 participants who switched to guselkumab 200 mg SC q4w from another treatment group and 9 participants who were already receiving guselkumab 200 mg SC q4w and received sham adjustment. Approximately half of participants achieved clinical response 16 weeks after dose adjustment (Table 2). Participants from the guselkumab 100 mg SC q8w or ustekinumab reference groups who dose-adjusted to guselkumab 200 mg SC q4w achieved higher levels of clinical response and clinical remission 16 weeks after the dose adjustment compared with sham adjustment. However, data interpretation is limited by the small sample size.

**Table 2:** Clinical remission and clinical response 16 weeks after dose adjustment in participants who dose-adjusted to GUS 200 mg q4w during the LTE.

	**PBO SC → GUS 200 mg SC q4w** ^a^	PBO IV → UST → GUS 200 mg SC q4w	GUS 100 mg SC q8w → GUS 200 mg SC q4w^b^	GUS 200 mg SC q4w → GUS 200 mg SC q4w^c^	UST → GUS 200 mg SC q4w^d^	Combined GUS 200 mg SC q4w
**Participants who were dose adjusted in the LTE**	2	10	7	9	7	35
**Clinical remission at 16 weeks post–dose adjustment**	1 (50.0)	6 (60.0)	3 (42.9)	2 (22.2)	3 (42.9)	15 (42.9)
**Clinical response at 16 weeks post–dose adjustment**	1 (50.0)	6 (60.0)	4 (57.1)	4 (44.4)	4 (57.1)	19 (54.3)

Values are n or n (%). Clinical remission is defined as CDAI score <150. Clinical response is defined as a ≥100-point reduction from baseline in CDAI score or CDAI score <150.

Abbreviations: CDAI, Crohn’s Disease Activity Index; GUS, guselkumab; IV, intravenous; LTE, long-term extension; PBO, placebo; q4w, every 4 weeks; q8w, every 8 weeks; SC, subcutaneous; UST, ustekinumab.

a Participants randomized to PBO SC who did not switch to UST at week 12 and met the inadequate response criteria, and so had a dose adjustment to GUS 200 mg SC q4w dosing during the LTE.

b Participants receiving the lower GUS 100 mg SC q8w maintenance dose who met the inadequate response criteria, and so had a dose adjustment to GUS 200 mg SC q4w dosing during the LTE.

c Participants who were already receiving the GUS 200 mg SC q4w maintenance dose and met the inadequate response criteria, and so received a “sham” dose adjustment to GUS 200 mg SC q4w dosing during the LTE.

d Participants randomized to UST at week 0 or switched to UST at week 12 who continued SC UST maintenance dosing in the maintenance period and had a dose adjustment to GUS 200 mg SC q4w during the LTE.

### Safety

The primary safety analysis set included 239 participants who received any guselkumab dose from week 0 through the end of the LTE, with an average follow-up of 155.9 weeks. During the entire study period of GALAXI 1, the exposure-adjusted number of AEs was 249.0 per 100 PY of follow-up (Table 3). The most commonly reported AEs in guselkumab-treated participants were nasopharyngitis (12.3 events per 100 PY of follow-up), injection site erythema (10.4 events per 100 PY), and COVID-19 (9.8 events per 100 PY). The majority of AEs were mild or moderate. The exposure-adjusted number of infections was 62.9 per 100 PY. Most infections were considered mild or moderate and did not result in discontinuation of study treatment. Numbers of SAEs, discontinuations due to AEs, and serious infections were low (10.1, 5.0, and 2.7 events per 100 PY, respectively) (Table 3).

**Table 3:** Key safety events from week 0 through the end of the LTE study in guselkumab-treated participants (full safety analysis set).

	Combined guselkumab (n = 239)^a^
**Average duration of follow-up, wk**	155.9
**Average exposure, number of administrations**	31.6
**Total PY of follow-up**	714.0
**Participants with ≥1 AE**	210 (87.9)
**AEs per 100 PY of follow-up**	249.0
**Most common AEs^b^ per 100 PY of follow-up**	
**Nasopharyngitis**	12.3
**Injection site erythema**	10.4
**COVID-19**	9.8
**Headache**	7.4
**Crohn’s disease**	7.0
**Upper respiratory tract infection**	6.3
**Pyrexia**	5.9
**Participants with ≥1 SAE**	55 (23.0)
**SAEs per 100 PY of follow-up**	10.1
**Participants with AEs leading to discontinuation**	34 (14.2)
**AEs leading to discontinuation per 100 PY of follow-up**	5.0
**Deaths**	2 (0.8)
**Participants with serious infections**	16 (6.7)
**Serious infections per 100 PY of follow-up**	2.7
**AEs of interest**	
**Clinically important hepatic disorders^c^**	5 (2.1)
**Malignancy excluding NMSC^d^**	5 (2.1)
**NMSC^e^**	2 (0.8)
**VTE**	3 (1.3)
**MACE^f^**	2 (0.8)
**Opportunistic infections**	1 (0.4)
**Active tuberculosis**	0
**Anaphylactic reactions**	0
**Serum sickness reactions**	0

Values are n (%) unless otherwise indicated.

Abbreviations: AE, adverse event; LTE, long-term extension; MACE, major adverse cardiovascular event; NMSC, nonmelanoma skin cancer; PY, patient-years; q4w, every 4 weeks; SAE, serious adverse event; SC, subcutaneous; VTE, venous thromboembolism.

a Participants receiving any guselkumab dose, either randomized to guselkumab (100 mg q8w or 200 mg q4w SC) or undergoing dose adjustment (to 200 mg SC q4w). Duration begins at the first dose of guselkumab and ends at the earlier time of study termination or the end of study. Events after the first dose of guselkumab are included. Includes events up to and after the time of dose adjustment to guselkumab 200 mg SC q4w. This column also includes 1 participant who was randomized to placebo, crossed over to ustekinumab at week 12, and inadvertently received one SC injection of guselkumab 100 mg at week 24.

b Incidence of >5 events per 100 PY of follow-up in guselkumab-treated participants.

c Clinically important hepatic disorders are defined as hepatic disorder AEs reported as SAEs or AEs leading to discontinuation of study intervention.

d Excludes basal cell carcinoma and squamous cell carcinoma.

e Includes only basal cell carcinoma and squamous cell carcinoma.

f Events meeting MACE criteria underwent clinical review by a blinded physician independent of the study team for final determination of inclusion.

All individual SAEs (per MedDRA preferred term) occurred in 2 or fewer individuals except for COVID-19 and ileus, which were each reported in 3 (1.3%) guselkumab-treated participants. The only individual AEs resulting in treatment discontinuation reported in >2 guselkumab-treated participants were Crohn’s disease (n = 5 [2.1%]) and hepatic enzyme increased (n = 3 [1.3%]). Two deaths (1 sudden death, 1 road traffic accident) were reported during the LTE (both in guselkumab-treated participants); the deaths were deemed unrelated to the study agent, per the investigator. No deaths were reported through week 48.^11^

Proportions of participants with AEs and event rates per 100 PY of follow-up for the ustekinumab reference group (n = 114; including placebo → ustekinumab or randomized to ustekinumab) are summarized in Table S6.

From week 0 through end of the study, frequencies of malignancies, VTE, MACE, and clinically important hepatic disorders were low (<3% of participants) in the combined guselkumab group (Table 3) and ustekinumab reference group (Table S6). Malignancies (including nonmelanoma skin cancer) were reported in 7 guselkumab-treated participants, including 5 during the LTE phase (1 participant each with pancreatic carcinoma and skin angiosarcoma in the guselkumab 100 mg SC q8w group; 1 participant each with prostate cancer, malignant melanoma, and basal cell carcinoma [considered a nonserious AE] in the guselkumab 200 mg SC q4w group). Events occurring prior to week 48 have been described previously.^11^^,^^12^ Among the 3 guselkumab-treated participants with VTEs reported at any time during the study, 1 case (pulmonary embolism) occurred during the LTE in a guselkumab 200 mg SC q4w group participant; the event was considered nonserious and not related to study intervention and did not result in discontinuation. One MACE (sudden death) was reported during the LTE (guselkumab 200 mg SC q4w group participant). Five guselkumab-treated participants experienced hepatic disorder SAEs or AEs leading to discontinuation, including 3 participants during the LTE (1 participant with liver function test increased; 2 participants with hepatic enzyme increased; all in the guselkumab 200 mg SC q4w group). All were AEs leading to treatment discontinuation that were confounded by medical history or concomitant medications or were concurrent with AEs associated with hepatic disorder. No cases of Hy’s law were reported. There were no cases of anaphylactic reactions or serum sickness reactions in guselkumab-treated participants during the study. One opportunistic infection (a nonserious AE of ophthalmic herpes zoster) was reported during the LTE in a guselkumab 200 mg SC q4w participant. This AE was considered treatment related but did not lead to treatment discontinuation. There were no cases of active tuberculosis in any treatment group.

No clinically meaningful effects of guselkumab on hematologic or chemistry laboratory parameters were observed.

### Pharmacokinetics and immunogenicity

Pharmacokinetic and immunogenicity results through weeks 12 and 48 have been reported elsewhere.^11^^,^^12^

Median serum trough concentrations of guselkumab were generally consistent between weeks 48 and 240 (or time of dose adjustment) within each dose group (Figure S8). The median serum trough concentration in the guselkumab 200 mg SC q4w treatment group (7.54 to 10.26 µg/mL) was approximately 8-fold that in the guselkumab 100 mg SC q8w treatment group (0.82 to 1.52 µg/mL). No clear or consistent trend for exposure-response was observed by serum guselkumab concentration quartiles at week 240 for the proportions of participants in clinical remission or endoscopic response (Figure S9).

Among participants who received any dose of guselkumab and had appropriate samples for antibody evaluation (n = 234), the proportion who were positive for ADAs to guselkumab remained low through end of study (n = 14 of 234 [6.0%]). Four participants had neutralizing antibodies. One ADA-positive participant was not evaluable for neutralizing antibodies. Because few participants had antibodies to guselkumab (n = 14), the relationship between efficacy and incidence of antibodies was not assessed. Ustekinumab immunogenicity data are reported in the Supplementary Appendix.

### Medical resource utilization

From week 0 through week 240, 6 (3.2%) of 185 participants in the combined guselkumab group had Crohn’s disease–related surgeries, 53 (28.6%) had disease-related emergency room visits or hospitalizations (including surgery-related hospitalizations), and 8 (4.3%) had Crohn’s disease–related procedures. Data for the ustekinumab reference group are described in the Supplementary Appendix.

## Discussion

This analysis of the LTE of the GALAXI 1 study reports efficacy and safety with up to 5 years of long-term guselkumab treatment, reflecting, to our knowledge, the longest treatment experience reported to date for IL-23p19 subunit inhibitors in individuals with moderately to severely active Crohn’s disease. These results support the durable efficacy and safety of guselkumab in participants who continued guselkumab maintenance treatment assigned at randomization (week 0).

Participant attrition is an inherent issue in the evaluation of any long-term clinical trial data, considering that participants discontinue due to not only treatment-related issues, but also unrelated issues such as family planning or geography. Overall, approximately half of guselkumab-randomized GALAXI 1 participants completed treatment (including the induction, maintenance, and LTE phases). Among those who entered the LTE at week 48, approximately 70% continued their assigned guselkumab regimen during the LTE. To address some of the known limitations associated with long-term data, including participant attrition, efficacy data were analyzed using 3 different approaches.

The as-observed analysis approach included participants who entered the LTE, remained on treatment without dose adjustment, and had data available at the specified time point, without applying treatment failure rules. NRI was applied to both the primary analysis set (ie, all participants randomized in GALAXI 1) and the LTE analysis set (ie, only those participants who entered LTE). The primary analysis approach provides the most conservative estimate of long-term outcomes, preserving the randomized comparison and approximating the likelihood that a patient initiating guselkumab will maintain clinical and endoscopic improvement over 5 years. Conversely, the LTE analysis estimates response rates among participants continuing guselkumab maintenance therapy after prior induction and maintenance, a population expected to persist on and benefit from treatment. Interpretation of results from the 3 approaches together provides a balanced and comprehensive assessment of the long-term performance of guselkumab. While as-observed analyses reflect outcomes among participants who continued treatment and remained in follow-up through the time of evaluation of efficacy, the NRI analyses provide an estimate of treatment effect accounting for treatment discontinuations among participants who received treatment in the LTE. Together, these approaches offer complementary perspectives on long-term guselkumab efficacy.

Endoscopic evaluations are important objective measures of disease activity and severity for guiding treatment targets and decision making.^4^ Participants receiving SC maintenance treatment in GALAXI 1 underwent yearly endoscopic assessments (ie, every 48 weeks). The week 48 analysis of GALAXI 1 showed high rates of endoscopic response and endoscopic remission among participants receiving guselkumab.^11^ Results from this LTE analysis showed persistent endoscopic benefit through week 240, with >70% and >50% of LTE participants in the guselkumab group achieving endoscopic response and endoscopic remission, respectively, at week 240 (as-observed analysis).

LTE participants who had an inadequate response between weeks 52 and 80 met dose adjustment eligibility criteria. These participants were excluded from as-observed efficacy analyses after the time point of dose adjustment and were considered nonresponders for the NRI analyses. Participants from the guselkumab 100 mg SC q8w treatment group and the ustekinumab group who switched to guselkumab 200 mg SC q4w achieved higher levels of clinical response and clinical remission 16 weeks after dose adjustment compared with sham adjustment; however, the number of participants who met dose-adjustment criteria was small (n = 35). These exploratory findings suggest that patients with inadequate response to ustekinumab may benefit from treatment with guselkumab and provide preliminary insight into how clinicians might approach dose optimization and switching strategies. The phase 3 GALAXI 2 and GALAXI 3 studies, which include a larger number of patients who undergo dose adjustment, will provide additional data to validate these patterns.

In clinically relevant subgroup analyses, guselkumab showed durable efficacy in achieving clinical remission and endoscopic outcomes in both BIO-naive and BIO-IR participants. It should be noted, however, that the subgroup sample sizes were limited.

Guselkumab was well tolerated through approximately 5 years, and no new safety concerns were identified in the LTE. Occurrences of SAEs, serious infections, discontinuations due to AEs, and AEs of special interest remained low through the end of the study. Overall, the guselkumab safety profile was consistent with the well-known profile across other indications, including ulcerative colitis, plaque psoriasis, and psoriatic arthritis.^7^^,^^8^ Few guselkumab-treated participants (n = 14) developed ADAs to guselkumab during the 5 years of follow-up. Discontinuations of study drug due to worsening of Crohn’s disease or lack of efficacy were few among participants treated with guselkumab during the LTE.

These 5-year results are consistent with analyses of GALAXI 1 at weeks 12 and 48, which demonstrated that participants randomized to guselkumab IV induction → SC maintenance regimens achieved high rates of clinical efficacy outcomes.^11^^,^^12^ Our results are also consistent with the short-term (12-week) and long-term (48-week) efficacy and safety of guselkumab demonstrated in the phase 3 GALAXI 2 and GALAXI 3 studies.^13^

While other IL-23p19 inhibitors, risankizumab^15^^,^^16^ and mirikizumab,^17^^,^^18^ have also demonstrated long-term clinical and endoscopic efficacy in participants with moderately to severely active Crohn’s disease during 2 to 3 years of treatment, study design and population differences limit cross-study comparisons.

These findings should be considered in the context of the GALAXI 1 study design and potential limitations. LTE participants were those who, per the investigator, were expected to benefit from remaining on guselkumab therapy. Participants entering the LTE may respond better to treatment than those who discontinued treatment prior to start of the LTE. The smaller sample in LTE may also limit interpretation of results. Participants were unblinded after the week 48 database lock and analysis, such that most participants were receiving open-label treatment during the LTE. Participants receiving ustekinumab who met inadequate response criteria were not eligible for dose intensification to ustekinumab q4w dosing.

Concomitant therapies for Crohn’s disease could be adjusted per investigator discretion during the LTE, which may have impacted efficacy. Corticosteroid initiation or dose increase after week 48 was not considered a treatment failure (in both as-observed and NRI analyses). However, most participants remained corticosteroid-free throughout the study (ie, <10% of participants in the guselkumab group initiated or increased corticosteroid use during the LTE). All guselkumab group participants in clinical remission at week 240 were also in corticosteroid-free clinical remission.

This LTE study was intended to evaluate guselkumab efficacy and safety over time and was not powered for any formal comparisons between treatment groups, including ustekinumab. Ustekinumab was included as a reference arm to contextualize the guselkumab data.

Strengths of the GALAXI 1 study include the long duration of follow-up for patients receiving an IL-23 inhibitor (ie, 5 years), with treat-through data mirroring continuous guselkumab treatment without rerandomization, which closely reflects real-world clinical practice and provides more clinically meaningful estimates of long-term outcomes. Comprehensive efficacy data were evaluated throughout the study, including clinical, endoscopic, histologic, and inflammatory biomarker endpoints.

Across multiple analytic approaches, guselkumab demonstrated durable clinical and endoscopic remission through approximately 5 years of total follow-up. As-observed results describe outcomes among participants who remained on treatment, while the NRI analyses provide a more conservative estimate that incorporates treatment discontinuations. Interpretation of these results together provides a balanced assessment of long-term guselkumab performance. The guselkumab safety profile was consistent with previously established findings, with no newly identified safety signals.^7^^,^^8^ These 5-year results from GALAXI 1 support a favorable and durable benefit-risk profile for long-term guselkumab maintenance therapy in adults with moderately to severely active Crohn’s disease.

## Supplementary Material

izag055_Supplementary_Data

## Data Availability

The Johnson & Johnson data sharing policy is available at https://innovativemedicine.jnj.com/our-innovation/clinical-trials/transparency. As noted at this site, trial data access requests can be submitted through the YODA (Yale Open Data Access) Project site (http://yoda.yale.edu).
