# Supplementary material for: Five-year efficacy and safety of guselkumab for moderately to severely active Crohn’s disease: Results from the phase 2 GALAXI 1 trial
Source: Inflamm Bowel Dis. 2026 May 1;32(9):1669–82. doi: 10.1093/ibd/izag055 (PMC13533210; doi:10.1093/ibd/izag055)
Supplement: Supplementary file 1 [file izag055_supplementary_data.zip › izag055_Supplementary_Data/Afzali_Supplementary_Appendix_R1.pdf]

***Supplementary Appendix to:* Five-Year Efficacy and Safety of Guselkumab for Moderately to Severely Active Crohn's Disease: Results from the Phase 2 GALAXI 1 Trial**

Anita Afzali, MD[1*], Silvio Danese, MD, PhD[2], Remo Panaccione, MD, FRCPC[3], David T. Rubin, MD[4], Bruce E. Sands, MD[5], Walter Reinisch, MD, PhD[6], Julián Panés, MD[7], Rian van Rampelbergh, MD, MSc[8], Nat A. Terry, MD, PhD[9], Leonardo Salese, MD[9], Marion L. Vetter, MD[9], Jacqueline Yee, MS[9], Christopher Corbett, MSc[10], Wilbert van Duijnhoven, MSc[8], Tadakazu Hisamatsu, MD, PhD[11], Jane M. Andrews, MBBS, PhD, FRACP[12], Geert R. D'Haens, MD, PhD[13], on behalf of the GALAXI 1 investigators

[1]Division of Gastroenterology and Hepatology, University of Cincinnati College of Medicine, Cincinnati, OH, USA; [2]Gastroenterology and Endoscopy, IRCCS Ospedale San Raffaele and University Vita-Salute San Raffaele Milano, Italy; [3]Inflammatory Bowel Disease Unit, University of Calgary, Calgary, AB, Canada; [4]University of Chicago Medicine, Inflammatory Bowel Disease Center, Chicago, IL, USA; [5]Icahn School of Medicine at Mount Sinai, New York, NY, USA; [6]Division of Gastroenterology & Hepatology, Medical University of Vienna, Vienna, Austria; [7]Hospital Clínic de Barcelona, IDIBAPS, CIBERehd, Barcelona, Spain; [8]Johnson & Johnson, Antwerp, Belgium; [9]Johnson & Johnson, Spring House, PA, USA; [10]Johnson & Johnson, High Wycombe, UK; [11]Department of Gastroenterology and Hepatology, Kyorin University, Tokyo, Japan; [12]Gastrointestinal Service, Surgery Program, Central Adelaide Local Health Network and University of Adelaide, Adelaide, SA, Australia; [13]Department of Gastroenterology, Amsterdam University Medical Centers, Amsterdam, The Netherlands

SUPPLEMENTARY METHODS

Concomitant Crohn's disease medications

Participants could maintain stable doses of permitted concomitant Crohn's disease therapies including 5-aminosalicylate (5-ASA) compounds, 6-mercaptopurine (6-MP), azathioprine (AZA), and methotrexate (MTX) through Week 48. Oral corticosteroids were to be maintained at stable doses through Week 12, with mandatory tapering thereafter if medically feasible. Participants could not initiate oral or rectal 5-ASA compounds, 6-MP, AZA, MTX, or oral, parenteral, or rectal corticosteroids (including budesonide and beclomethasone dipropionate) through Week 48. During the long-term extension (LTE), concomitant therapies for Crohn's disease could be adjusted at the discretion of the investigator.

SUPPLEMENTARY RESULTS

Immunogenicity: ustekinumab reference group

In the ustekinumab reference group, 1/114 (0.9%) participant was positive for antibodies to ustekinumab (also neutralizing).

Medical resource utilization: ustekinumab reference group

Among ustekinumab-randomized participants in the reference arm, 1/63 (1.6%) had a Crohn's disease-related surgery, 15 (23.8%) had Crohn's disease-related emergency room visits or hospitalizations (including surgeries), and 2 (3.2%) had Crohn's disease-related procedures.

Supplementary Table 1. Definitions of efficacy endpoints evaluated during the LTE

Endpoint	Definition
Clinical remission	CDAI <150
Durable clinical remission	CDAI <150 for at least 20 of 25 visits from Week 48 through Week 240, including Week 240
Corticosteroid-free clinical remission	CDAI <150 at landmark timepoints and not having received corticosteroids for at least 90 days prior to the timepoint
PRO-2 remission	AP mean daily score ≤1 and SF mean daily score ≤3, and no worsening of AP or SF from baseline
Endoscopic response	≥50% improvement from baseline in SES-CD or SES-CD ≤2
Endoscopic remission	SES-CD ≤4 with at least a 2-point reduction from baseline and no subscore >1 in any subcomponent
Clinical remission *and* endoscopic response	Participant-level composite of clinical remission and endoscopic response
Deep remission	Participant-level composite of clinical remission and endoscopic remission
Histologic response (RHI)	≥50% reduction in RHI score from baseline or a score ≤3 with sub-scores of lamina propria neutrophils and neutrophils in epithelium equal to 0, with no ulcers or erosions, among participants with histologic disease activity at baseline (with histologic disease activity defined as a score >0 for any of items 2-4 of the RHI [lamina propria neutrophils, neutrophils in epithelium, or erosions or ulcerations])
Histo-endoscopic response	Participant-level composite of histologic response and endoscopic response
CRP normalization	CRP ≤3 mg/L among participants with abnormal CRP (>3 mg/L) at baseline
Fecal calprotectin normalization	Fecal calprotectin ≤250 µg/g among participants with abnormal fecal calprotectin (>250 µg/g) at baseline

AP=abdominal pain; CDAI=Crohn's Disease Activity Index; CRP=C-reactive protein; LTE=long-term extension; PRO-2= patient-reported outcome-2; RHI=Robarts Histopathology Index; SES-CD=Simple Endoscopic Score for Crohn's Disease; SF=stool frequency.

Supplementary Table 2. Statistical analysis methods

	1. As-observed	2. LTE NRI	3. Primary efficacy analysis set
**Analysis population**	LTE efficacy analysis set of randomized participants who entered the LTE phase and remained on continuous treatment at the specific timepoint of analysis	LTE analysis set of randomized participants who entered the LTE phase and received ≥1 dose (full or partial) of study treatment	Primary efficacy analysis set of participants who were randomized at Week 0 (baseline) and received ≥1 dose (full or partial) of study drug, regardless of whether or not they entered the LTE[a]
**Randomized participants who did not enter the LTE**	Excluded from analysis	Excluded from analysis	Considered non-responders throughout the LTE period
**Treatment failure rules**			
Crohn's disease-related surgery or discontinued study agent due to lack of efficacy or an AE of worsening Crohn's disease prior to the designated analysis timepoint	*Observed data; no treatment failure rules applied*	Considered non-responders from that timepoint onwards	Considered non-responders from that timepoint onwards
Discontinued study agent due to reasons other than COVID-19 restrictions/issues, lack of efficacy, or AE of worsening Crohn's disease prior to the designated analysis timepoint		Observed data used from that timepoint onwards, if available, to determine responder and non-responder status	Observed data used from that timepoint onwards, if available, to determine responder and non-responder status
Discontinued study agent due to COVID-19 restrictions/issues prior to the designated analysis timepoint		Did not have their data used from that timepoint onwards	Did not have their data used from that timepoint onwards

	1. As-observed	2. LTE NRI	3. Primary efficacy analysis set
**Medication changes and dose adjustment**			
Dose adjustment at any visit from Week 52 to Week 80	Excluded after the time of dose adjustment	Considered non-responders from that visit onwards	Considered non-responders from that visit onwards
Prohibited changes in Crohn's disease medications prior to Week 48[b]	N/A	N/A	Considered non-responders from that visit onwards
**Handling of missing data**			
Missing endpoint data at a designated analysis timepoint	Excluded from analysis at that visit	Considered non-responders at the timepoint (after applying above treatment failure rules)	Considered non-responders at the timepoint (after applying above treatment failure rules)

Note: Participants who completed the study at Week 144 under an earlier version of the protocol are excluded from the denominator for all analyses thereafter.

[a] Excluding participants whose induction dosing was discontinued as a result of an urgent safety measure [N=51].

[b] Changes to concomitant Crohn's disease medications after Week 48 were not considered treatment failures.

AE=adverse event; COVID-19=coronavirus disease 2019; LTE=long-term extension, N/A=not applicable, NRI=non-responder imputation.

Supplementary Table 3. Demographics and disease characteristics of the ustekinumab reference group at baseline (Week 0) of GALAXI 1 (LTE participants)

	Ustekinumab Reference Arm[a] (N=48)
Age, years	
Mean (SD)	34.9 (12.3)
Median (IQR)	34.5 (24.0, 41.5)
Sex, n (%)	
Men	32 (66.7)
Women	16 (33.3)
Race, n (%)	
American Indian or Alaska Native	0
Asian	3 (6.3)
Black or African American	1 (2.1)
Native Hawaiian or other Pacific Islander	0
White	44 (91.7)
Not reported	0
Ethnicity, n (%)	
Hispanic or Latino	0
Not Hispanic or Latino	47 (97.9)
Not reported	1 (2.1)
Mean (SD) weight, kg	69.1 (16.4)
CD duration, years	
Mean (SD)	7.5 (6.3)
Median (IQR)	5.9 (2.5, 10.5)
CDAI score, mean (SD)	307.3 (61.0)
Median (IQR)	289.5 (259.0, 354.0)
PRO-2 score, mean (SD)	143.9 (42.1)
Stool frequency >3, n (%)	43 (89.6)
Abdominal pain >1, n (%)	46 (95.8)
SES-CD, mean (SD)	14.3 (8.0)
Median (IQR)	14.5 (7.0, 19.5)
CRP concentration, mg/L	
Mean (SD)	16.4 (26.9)
Median (IQR)	6.9 (1.8, 16.9)
Fecal calprotectin, µg/g	
Mean (SD)	1380.9 (1561.8)
Median (IQR)	675.5 (211.5, 1818.5)
IBDQ score, mean (SD)	130.3 (31.1)
Endoscopic disease severity per SES-CD, n (%)	
Moderate (7-16)	19 (39.6)
Severe (>16)	19 (39.6)
Involved GI areas by central read, n (%)	
Ileum only	9 (18.8)
Colon only	22 (45.8)
Ileum and colon	17 (35.4)

	Ustekinumab Reference Arm[a] (N=48)
≥1 open or draining fistula, n (%)	9 (18.8)
Prior and concomitant Crohn's disease medications	
≥1 Crohn's disease medications taken at baseline, n (%)	38 (79.2)
Immunomodulatory drugs	19 (39.6)
Corticosteroids, including budesonide and beclomethasone dipropionate	18 (37.5)
Corticosteroids, excluding budesonide and beclomethasone dipropionate	13 (27.1)
Median (IQR) prednisone-equivalent dose, mg/day[b]	22.7 (12.8)
Prior inadequate response or intolerance to biologic, n (%)	25 (52.1)
≥1 anti-TNF[c]	25 (52.1)
≥2 anti-TNF	7 (14.6)
Anti-TNF only	23 (47.9)
Vedolizumab	2 (4.2)
Vedolizumab and ≥1 anti-TNF	2 (4.2)
Biologic-naïve, n (%)	16 (33.3)
Biologic experienced without documented inadequate response or intolerance, n (%)	7 (14.6)

[a] Includes only participants who were randomized to ustekinumab at Week 0.

[b] Excluding budesonide and beclomethasone dipropionate.

[c] Anti-TNF includes adalimumab (n=12), infliximab (n=18), and certolizumab pegol (n=4).

CD=Crohn's disease; CDAI=Crohn's Disease Activity Index; CRP=C-reactive protein; GI=Gastrointestinal; IBDQ=Inflammatory Bowel Disease Questionnaire; IQR=Interquartile range; LTE=Long-term extension; PRO-2=Patient-reported outcome-2; SD=Standard deviation; SES-CD=Simple Endoscopic Score for Crohn's Disease; TNF=Tumor necrosis factor.

Supplementary Table 4. Crohn's disease characteristics at Week 48 for the combined guselkumab group (LTE participants)

	Combined Guselkumab[a] (N=151)
CDAI score, mean (SD)	87.9 (70.8)
Median (IQR)	76.0 (27.0, 128.0)
PRO-2 score, mean (SD)	36.0 (36.5)
Stool frequency >3, n (%)	22 (14.6)
Abdominal pain >1, n (%)	20 (13.2)
SES-CD, mean (SD)	5.2 (5.0)[b]
Median (IQR)	4.0 (0.0, 7.0)
CRP concentration, mg/L	
Mean (SD)	4.9 (9.5)[c]
Median (IQR)	1.9 (0.7, 4.2)[c]
Fecal calprotectin, µg/g	
Mean (SD)	527.9 (2246.8)[d]
Median (IQR)	121.5 (47.0, 328.5)[d]
IBDQ score, mean (SD)	180.9 (29.2)[e]
Endoscopic disease severity per SES-CD, n (%)	
Moderate (7-16)	42 (28.8)[b]
Severe (>16)	5 (3.4)[b]
Involved GI areas by central read, n (%)	
Ileum only	36 (24.7)[b]
Colon only	53 (36.3)[b]
Ileum and colon	20 (13.7)[b]
≥1 open or draining fistula, n (%)	16 (10.6)

[a] Includes all participants receiving either guselkumab 100 mg SC q8w or guselkumab 200 mg SC q4w at start of the LTE.
[b] N=146.
[c] N=150.
[d] N=136.
[e] N=148.
CDAI=Crohn's Disease Activity Index; CRP=C-reactive protein; GI=Gastrointestinal; IBDQ=Inflammatory Bowel Disease Questionnaire; IQR=Interquartile range; LTE=Long-term extension; PRO-2=Patient-reported outcome-2; q4w=Every 4 weeks; q8w=Every 8 weeks; SC=Subcutaneous; SD=Standard deviation; SES-CD=Simple Endoscopic Score for Crohn's Disease.

Supplementary Table 5. Summary of average daily prednisone-equivalent corticosteroid dose (mg/day) by visit among LTE participants who were receiving corticosteroids at baseline, in the combined guselkumab group[a]

	Combined Guselkumab[b]
Baseline	
N	33
Mean (SD)	22.50 (11.354)
Median	20.00
IQR	20.0, 25.0
Range	(5.0; 60.0)
Week 48	
N	33
Mean (SD)	3.02 (7.093)
Median	0.00
IQR	0.0, 0.0
Range	(0.0; 30.0)
Week 96	
N	31
Mean (SD)	1.13 (3.810)
Median	0.00
IQR	0.0, 0.0
Range	(0.0; 20.0)
Week 144	
N	30
Mean (SD)	1.00 (3.806)
Median	0.00
IQR	0.0, 0.0
Range	(0.0; 20.0)
Week 192	
N	29
Mean (SD)	1.90 (6.603)
Median	0.00
IQR	0.0, 0.0
Range	(0.0; 30.0)
Week 240	
N	28
Mean (SD)	0.89 (3.862)
Median	0.00
IQR	0.0, 0.0
Range	(0.0; 20.0)

[a] Excluding budesonide and beclomethasone dipropionate.
[b] Includes all participants receiving either guselkumab 100 mg SC q8w or guselkumab 200 mg SC q4w. Participants who completed the study at Week 144 under a previous version of the protocol are excluded at timepoints after Week 144.

Handling of missing data: participants who had a Crohn's disease-related surgery, or discontinued study agent due to lack of efficacy or an AE of worsening Crohn's disease prior to the designated analysis timepoint had their highest dose in the last two weeks carried forward from that timepoint onwards. Participants who had discontinued study agent due to the reasons other than COVID-19 restrictions/issues, lack of efficacy or AE of worsening Crohn's disease prior to the designated analysis timepoint had their observed data used, if available at that timepoint. Participants who had discontinued study agent due to COVID-19 restrictions/issues prior to the designated analysis timepoint did not have their data used from that timepoint onwards. Participants who had a dose adjustment at any visit from Week 52 to Week 80 had their highest dose in the last two weeks carried forward from that timepoint onwards. After applying these rules, participants who had a missing value in corticosteroid use at the designated analysis timepoint did not have their missing data imputed at that timepoint.
AE=Adverse event; COVID-19=Coronavirus disease 2019; IQR=Interquartile range; LTE=Long-term extension; q4w=Every 4 weeks; q8w=Every 8 weeks; SC=Subcutaneous; SD=Standard deviation.

Supplementary Table 6. Key safety events from Week 0 through end of study in ustekinumab reference group (full safety analysis set)

	Ustekinumab[a] (N=114)
Average duration of follow-up, weeks	150.0
Average exposure, number of administrations	17.8
Total PY of follow-up	327.8
Participants with ≥1 AE, n (%)	102 (89.5)
AEs per 100 PY of follow-up	184.0
Most common AEs[b] per 100 PY of follow-up	
Crohn's disease	13.1
Anemia	7.9
Upper respiratory tract infection	7.6
Arthralgia	5.8
Nasopharyngitis	5.8
Abdominal pain	5.2
COVID-19	5.2
Participants with ≥1 SAE, n (%)	18 (15.8)
SAEs per 100 PY of follow-up	10.4
Participants with AEs leading to discontinuation, n (%)	12 (10.5)
AEs leading to discontinuation per 100 PY of follow-up	4.3
Deaths, n (%)	0
Participants with serious infections, n (%)	6 (5.3)
Serious infections per 100 PY of follow-up	2.7
AEs of interest, n (%) of participants	
Anaphylactic reactions	1 (0.9)
Clinically important hepatic disorders[c]	1 (0.9)
Malignancy excluding NMSC[d]	1 (0.9)
NMSC[e]	0
Active tuberculosis	0
MACE[f]	0
Opportunistic infections	0
Serum sickness reactions	0
VTE	0

[a] Includes placebo → ustekinumab and ustekinumab ~6 mg/kg IV → 90 mg SC q8w group participants. Includes events up to the time of dose adjustment (change in treatment, change in dose, or sham adjustment).
[b] Incidence of >5 events per 100 PY of follow-up in ustekinumab-treated participants.
[c] Clinically important hepatic disorders are defined as hepatic disorder AEs reported as SAEs or AEs leading to discontinuation of study intervention.
[d] Excludes basal cell and squamous cell carcinoma.
[e] Includes only basal cell and squamous cell carcinoma.
[f] Events meeting MACE criteria underwent clinical review by a blinded physician independent of the study team for final determination of inclusion.
AE=Adverse event; COVID-19=Coronavirus disease 2019; IV=Intravenous; MACE=Major adverse cardiovascular event; NMSC=Nonmelanoma skin cancer; PY=Patient-years; q8w=Every 8 weeks; SAE=Serious adverse event; SC=Subcutaneous; VTE=Venous thromboembolism.

Supplementary Figure 1. Durable clinical remission (A) and corticosteroid-free (90 days) clinical remission (B) in LTE participants in the combined guselkumab group

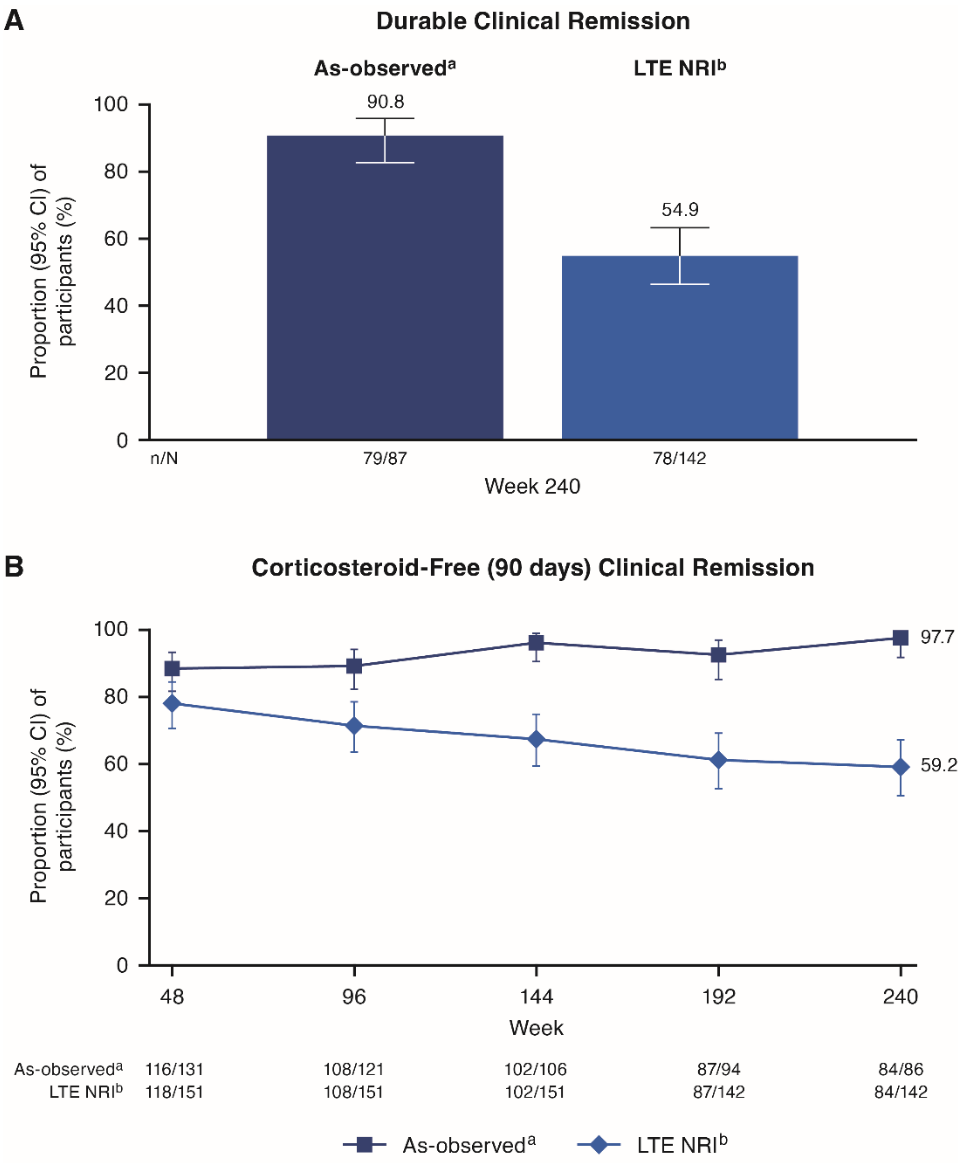

[a] Observed rates were evaluated in participants who entered the LTE and remained on treatment at that specific timepoint. Participants who dose-adjusted were excluded after the time of dose adjustment. No treatment failure rules were applied. Participants who did not have data at a visit were excluded from analysis for that visit.

[b] Participants who had a Crohn's disease-related surgery, or discontinued study agent due to lack of efficacy or an AE of worsening Crohn's disease prior to the designated analysis timepoint were considered non-responders from that timepoint onwards. Participants who had discontinued study agent due to reasons other than COVID-19 restrictions/issues, lack of efficacy or AE of worsening Crohn's disease prior to the designated analysis timepoint had their observed data used, if available, to determine responder and non-responder status from that timepoint onwards. Participants who had discontinued study agent due to COVID-19 restrictions/issues prior to the designated analysis timepoint did not have their data used from that timepoint onwards. Participants who had a dose adjustment at any visit from Week 52 to Week 80 were considered non-responders from that visit onwards. After applying these rules, participants with missing data at the designated analysis timepoint were considered non-responders at that timepoint.

Participants who completed the study at Week 144 under a previous version of the protocol were excluded from the denominator for analyses at timepoints after Week 144.
Clinical remission is defined as CDAI score <150. Durable clinical remission in the LTE is defined as CDAI score <150 for at least 20 of 25 visits (i.e., ≥80% of visits) from Week 48 through Week 240 which must include Week 240.
Corticosteroid-free (90 days) clinical remission is defined as CDAI score <150 and not receiving corticosteroids for at least 90 days prior to that timepoint. CIs based on the exact method. Participants who completed the study at Week 144 under an earlier version of the protocol are excluded from the denominator for all analyses thereafter.
AE=Adverse event; CDAI=Crohn's Disease Activity Index; CI=Confidence interval; COVID-19=Coronavirus disease 2019; CS=Corticosteroid; LTE=Long-term extension; NRI=Non-responder imputation.

Supplementary Figure 2. Clinical remission *and* endoscopic response (A) and deep remission (B) by visit through Week 240 in the combined guselkumab group

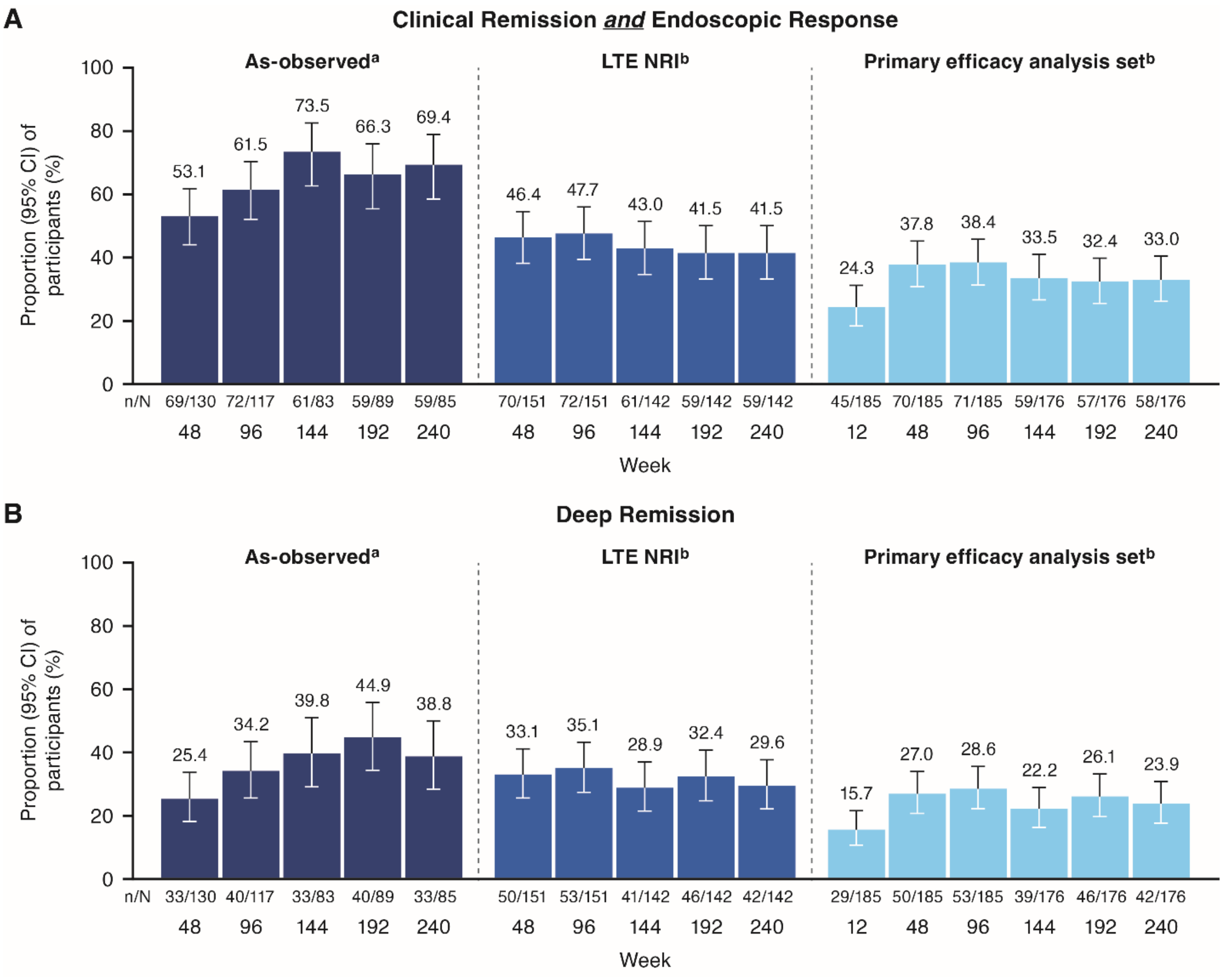

[a] Observed rates were evaluated in participants who entered the LTE and remained on treatment at that specific timepoint. Participants who dose-adjusted were excluded after the time of dose adjustment. No treatment failure rules were applied. Participants who did not have data at a visit were excluded from analysis for that visit.

[b] Participants with missing data at a particular timepoint after accounting for intercurrent event strategies were considered not to have achieved the endpoint at that timepoint.

Participants who completed the study at Week 144 under an earlier version of the protocol were not scheduled to have an ileocolonoscopy from Week 144 and were excluded from analysis at Week 144 and thereafter**.**

Clinical remission is defined as CDAI score <150; endoscopic response defined as ≥50% improvement from baseline in SES-CD or SES-CD ≤2. Endoscopic response is defined as is defined as ≥50% improvement from baseline in SES-CD or SES-CD ≤2. Deep remission is defined as achieving both clinical remission (CDAI score <150) and endoscopic remission (defined as SES-CD ≤4 with at least a 2-point reduction from baseline and no subscore >1 in any subcomponent).

CDAI=Crohn's Disease Activity Index; CI=Confidence interval; LTE=Long-term extension; NRI=Non-responder imputation; SES-CD=Simple Endoscopic Score for Crohn's Disease.

Supplementary Figure 3. IBDQ remission rates in the combined guselkumab group from Week 48 through Week 240 (LTE participants; NRI analysis).

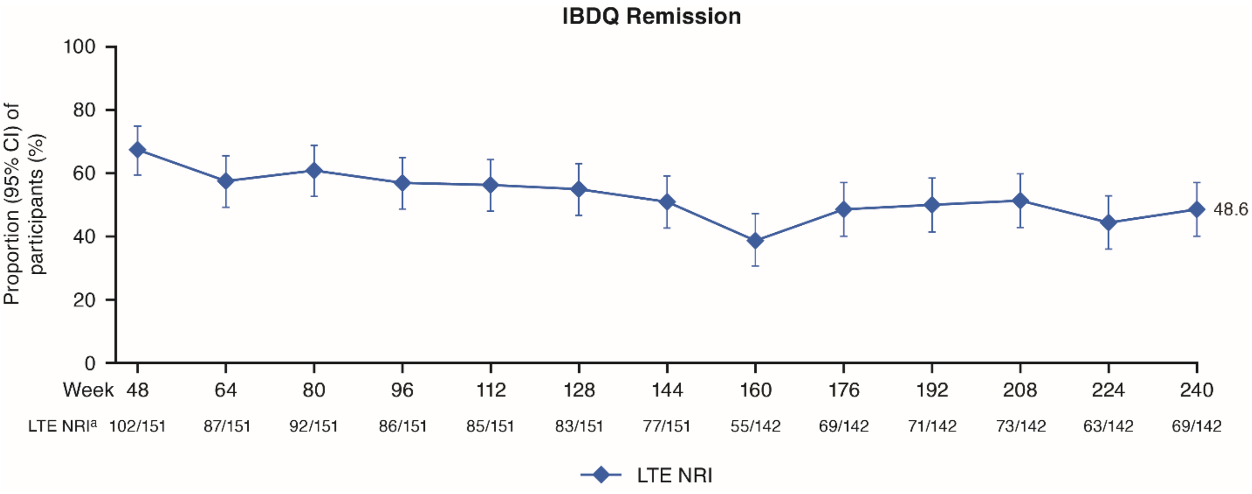

[a] Participants who completed the study at Week 144 under a previous version of the protocol are excluded from the denominator at Week 160 and after. Participants who had a Crohn's disease-related surgery or discontinued study agent due to lack of efficacy or an AE of worsening Crohn's disease prior to the designated analysis timepoint were considered not to be in IBDQ remission from that timepoint onwards. Participants who had discontinued study agent due to reasons other than COVID-19 restrictions/issues, lack of efficacy or AE of worsening Crohn's disease prior to the designated analysis timepoint had their observed data used, if available, to determine responder and non-responder status from that timepoint onwards. Participants who had discontinued study agent due to COVID-19 restrictions/issues prior to the designated analysis timepoint did not have their data used from that timepoint onwards. Participants who had a dose adjustment at any visit from Week 52 to Week 80 were considered not to be in IBDQ remission from that visit onwards. After applying these intercurrent event rules, participants who had missing data at the designated analysis timepoint were considered not to be in IBDQ remission at that timepoint. CIs were based on the exact method.

IBDQ remission is defined as IBDQ score ≥170.

AE=adverse event; CI=Confidence interval; COVID-19=Coronavirus disease 2019; IBDQ=Inflammatory Bowel Disease Questionnaire; LTE=Long-term extension; NRI=Non-responder imputation.

Supplementary Figure 4. Histologic response (A) and histo-endoscopic response (B) by study visit in the combined guselkumab group (LTE participants; RHI histology assessment)

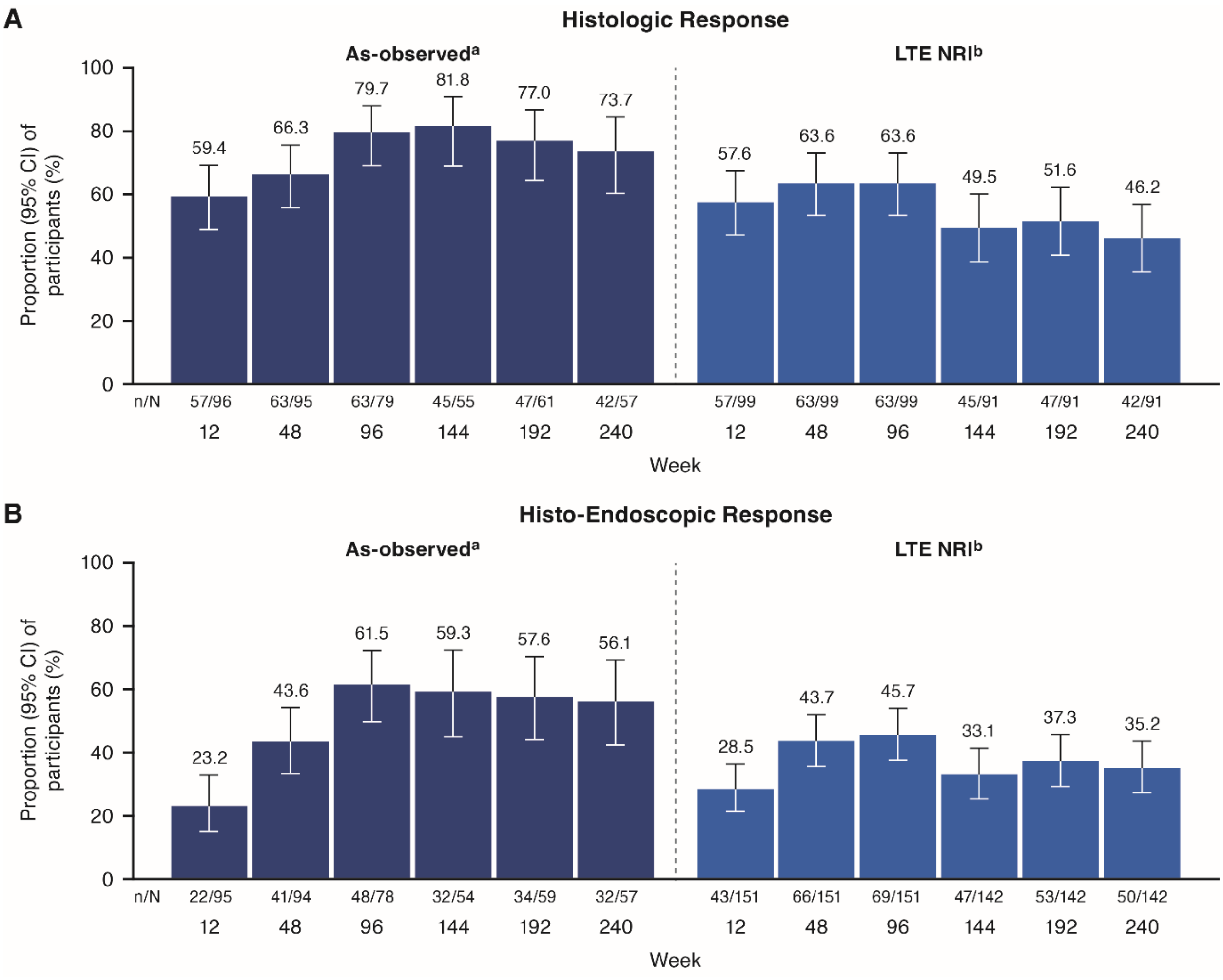

[a] Observed rates evaluated in participants who entered the LTE and remained on treatment at that specific timepoint. Participants who dose adjusted were excluded after the time of dose adjustment. No treatment failure rules were applied. Participants who did not have data at a visit were excluded from analysis for that visit.

[b] Participants who had a Crohn's disease-related surgery or discontinued study agent due to lack of efficacy or an AE of worsening Crohn's disease prior to the designated analysis timepoint were considered non-responders from that timepoint onwards. Participants who had discontinued study agent due to reasons other than COVID-19 restrictions/issues, lack of efficacy, or AE of worsening Crohn's disease prior to the designated analysis timepoint had their observed data used, if available, to determine responder and non-responder status from that timepoint onwards. Participants who had discontinued study agent due to COVID-19 restrictions/issues prior to the designated analysis timepoint did not have their data used from that timepoint onwards. Participants who had a dose adjustment at any visit from Week 52 to Week 80 were considered non-responders from that visit onwards. After applying these rules, participants with missing data at the designated analysis timepoint were considered non-responders at that timepoint (NRI).

Participants who completed the study at Week 144 under a previous version of the protocol are excluded from the denominator for all analyses thereafter (and Week 144 for endoscopic analyses).

Histologic response is defined as ≥50% reduction in RHI score from baseline or a score ≤3, sub-scores of lamina propria neutrophils and neutrophils in epithelium equal to 0, with no ulcers or erosions. Baseline histologic disease activity is defined as a score >0 for any of Items 2-4 of RHI (lamina propria neutrophils, neutrophils in epithelium,

or erosions or ulcerations). Histologic response was evaluated in participants with histologic disease activity at baseline (score >0 for any of Items 2-4 of RHI [lamina propria neutrophils, neutrophils in epithelium, or erosions or ulcerations]. Histo-endoscopic response is defined as a combination of histologic response (per indicated definition) and endoscopic response (≥50% improvement from baseline in SES-CD or SES-CD ≤2).
AE=Adverse event; CI=Confidence interval; COVID-19=Coronavirus disease 2019; LTE=Long-term extension; NRI=Non-responder imputation; RHI=Robarts Histopathology Index; SES-CD=Simple Endoscopic Score for Crohn's Disease.

Supplementary Figure 5. Summary of inflammatory biomarker endpoints in the combined guselkumab group. (A) CRP normalization (≤3 mg/L) among participants with CRP >3 mg/L at baseline. (B) Fecal calprotectin normalization (≤250 µg/g) among participants with fecal calprotectin >250 µg/g at baseline.

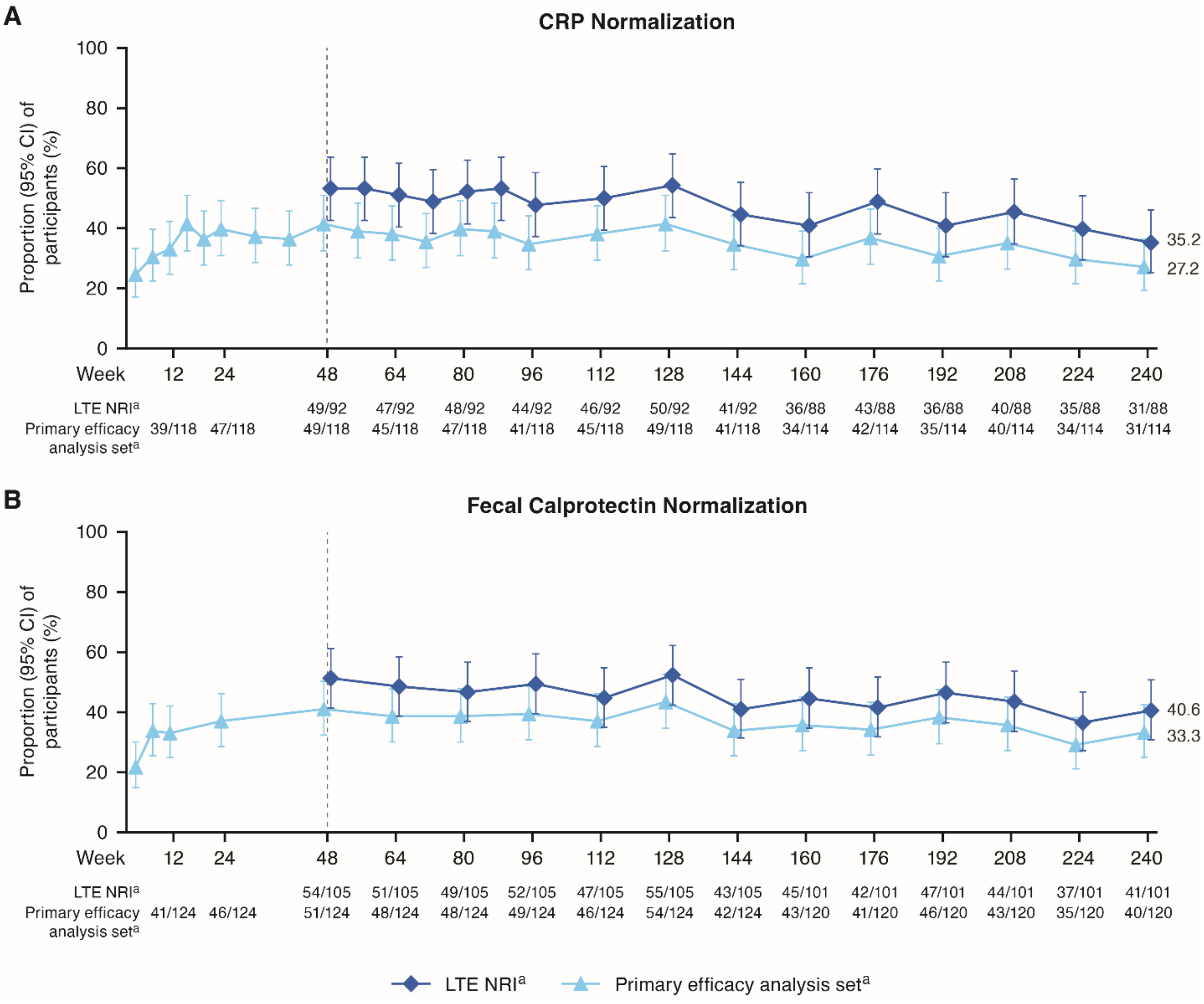

[a] Participants who had a Crohn's disease-related surgery, or discontinued study agent due to lack of efficacy or an AE of worsening Crohn's disease prior to the designated analysis timepoint were considered non-responders from that timepoint onwards. Participants who had discontinued study agent due to reasons other than COVID-19 restrictions/issues, lack of efficacy or AE of worsening Crohn's disease prior to the designated analysis timepoint had their observed data used, if available, to determine responder and non-responder status from that timepoint onwards. Participants who had discontinued study agent due to COVID-19 restrictions/issues prior to the designated analysis timepoint did not have their data used from that timepoint onwards. Participants who had a dose adjustment at any visit from Week 52 to Week 80 were considered non-responders from that visit onwards. After applying these rules, participants with missing data at the designated analysis timepoint were considered non-responders at that timepoint (NRI).

Participants who completed the study at Week 144 under an earlier version of the protocol are excluded from the denominator for all analyses thereafter.

AE=Adverse event; CI=Confidence interval; COVID-19=Coronavirus disease 2019; CRP=C-reactive protein; LTE=Long-term extension; NRI=Non-responder imputation.

Supplementary Figure 6. Clinical and endoscopic outcomes by visit in subgroups of the combined guselkumab group by BIO-naïve or BIO-IR.

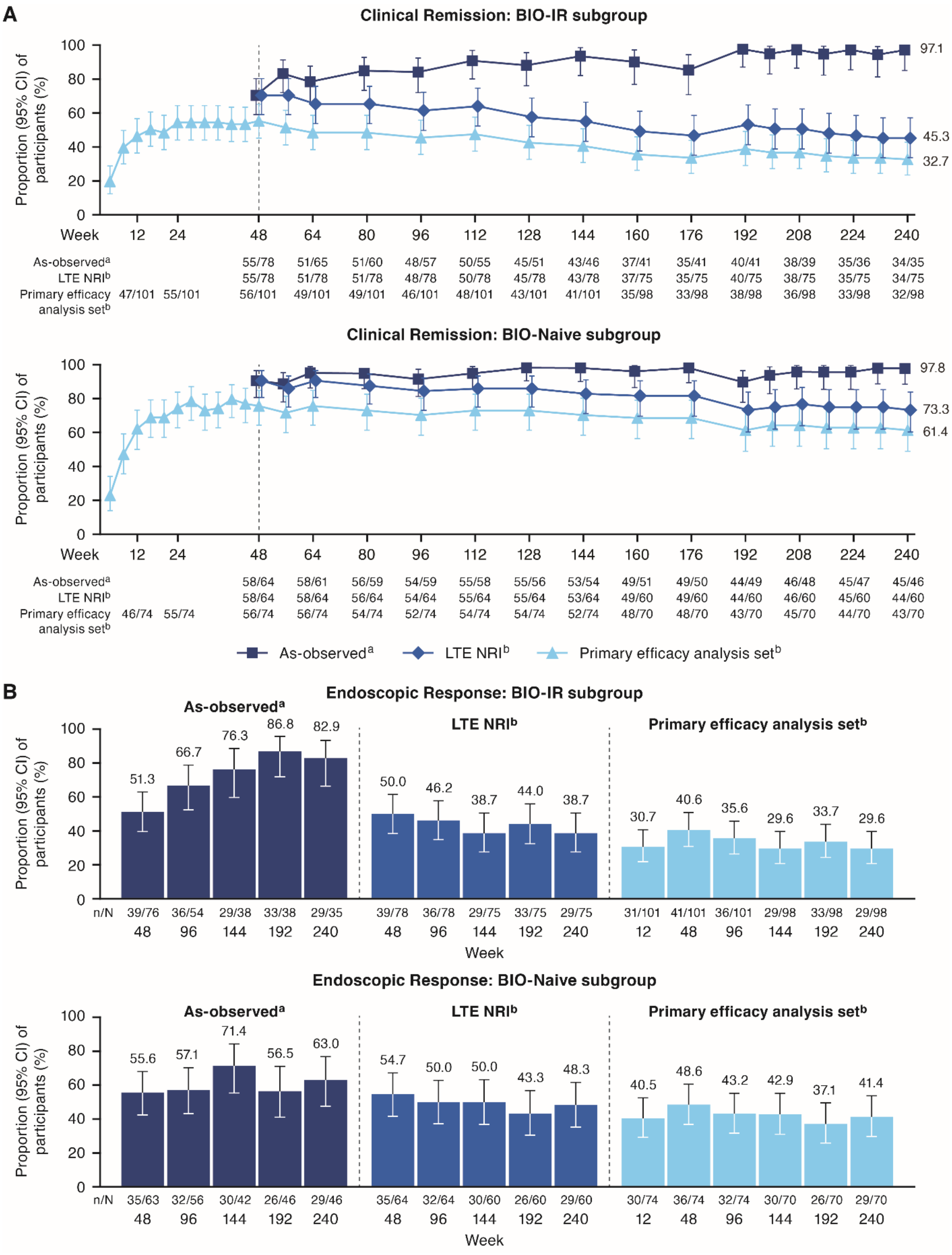

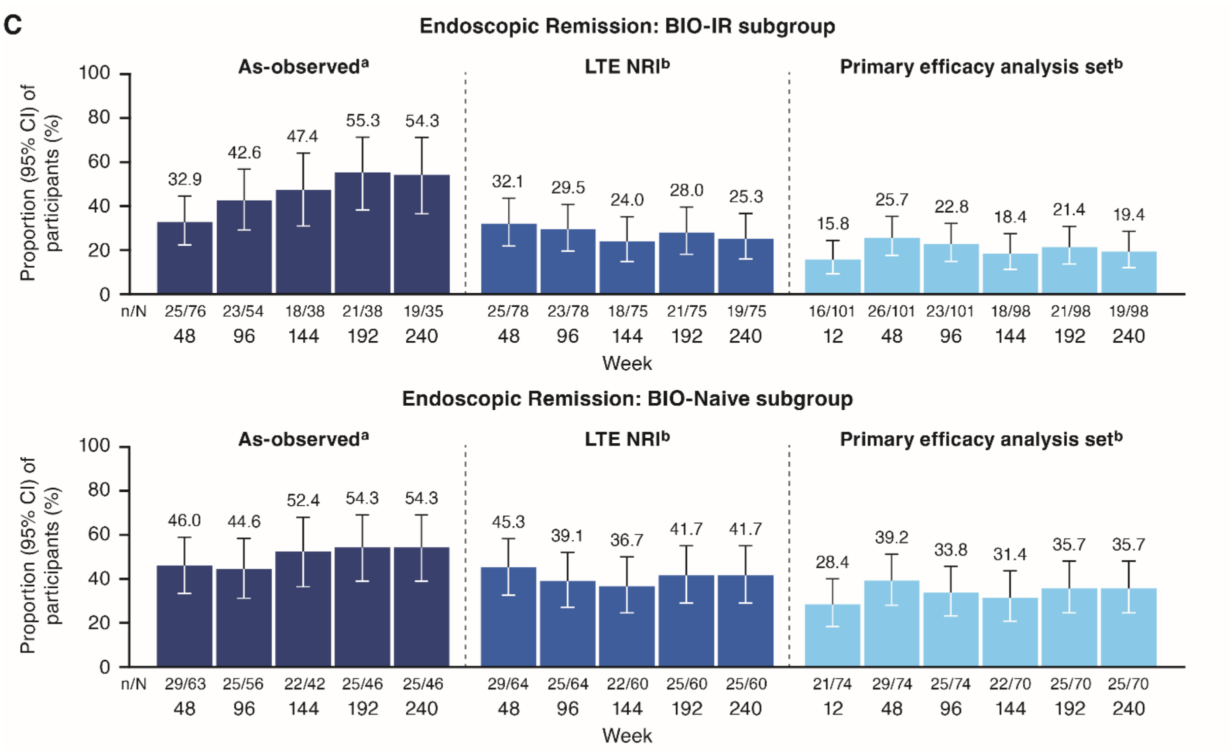

[a] Observed rates evaluated during the first four years of the LTE in participants who entered the LTE and remained on treatment at that specific timepoint. Participants who dose adjusted were excluded after the time of dose adjustment. No treatment failure rules were applied. Participants who did not have data at a visit were excluded from analysis for that visit.

[b] Participants who had a Crohn's disease-related surgery, or discontinued study agent due to lack of efficacy or an AE of worsening Crohn's disease prior to the designated analysis timepoint were considered non-responders from that timepoint onwards. Participants who had discontinued study agent due to reasons other than COVID-19 restrictions/issues, lack of efficacy or AE of worsening Crohn's disease prior to the designated analysis timepoint had their observed data used, if available, to determine responder and non-responder status from that timepoint onwards. Participants who had discontinued study agent due to COVID-19 restrictions/issues prior to the designated analysis timepoint did not have their data used from that timepoint onwards. Participants who had a dose adjustment at any visit from Week 52 to Week 80 were considered non-responders from that visit onwards. After applying these rules, participants with missing data at the designated analysis timepoint were considered non-responders at that timepoint (NRI).

Participants who completed the study at Week 144 under a previous version of the protocol are excluded from the denominator for analyses at timepoints after Week 144. These participants were not scheduled to have an ileocolonoscopy at Week 144 and were excluded from analysis of endoscopic response and endoscopic remission at Week 144 and thereafter.

Clinical remission is defined as CDAI score <150. Endoscopic response is defined as ≥50% improvement from baseline in SES-CD or SES-CD ≤2. Endoscopic remission is defined as SES-CD ≤ 4 with at least a 2-point reduction from baseline and no subscore greater than 1 in any individual subcomponent.

AE=Adverse event; BIO-IR=History of inadequate response or intolerance to biologic therapy; BIO-naïve=Naïve to biologic therapy; CDAI=Crohn's Disease Activity Index; CI=Confidence interval; COVID-19=Coronavirus disease 2019; LTE=Long-term extension; NRI=Non-responder imputation; SES-CD=Simple Endoscopic Score for Crohn's Disease.

Supplementary Figure 7. Clinical and endoscopic endpoints by visit in the ustekinumab-randomized reference group

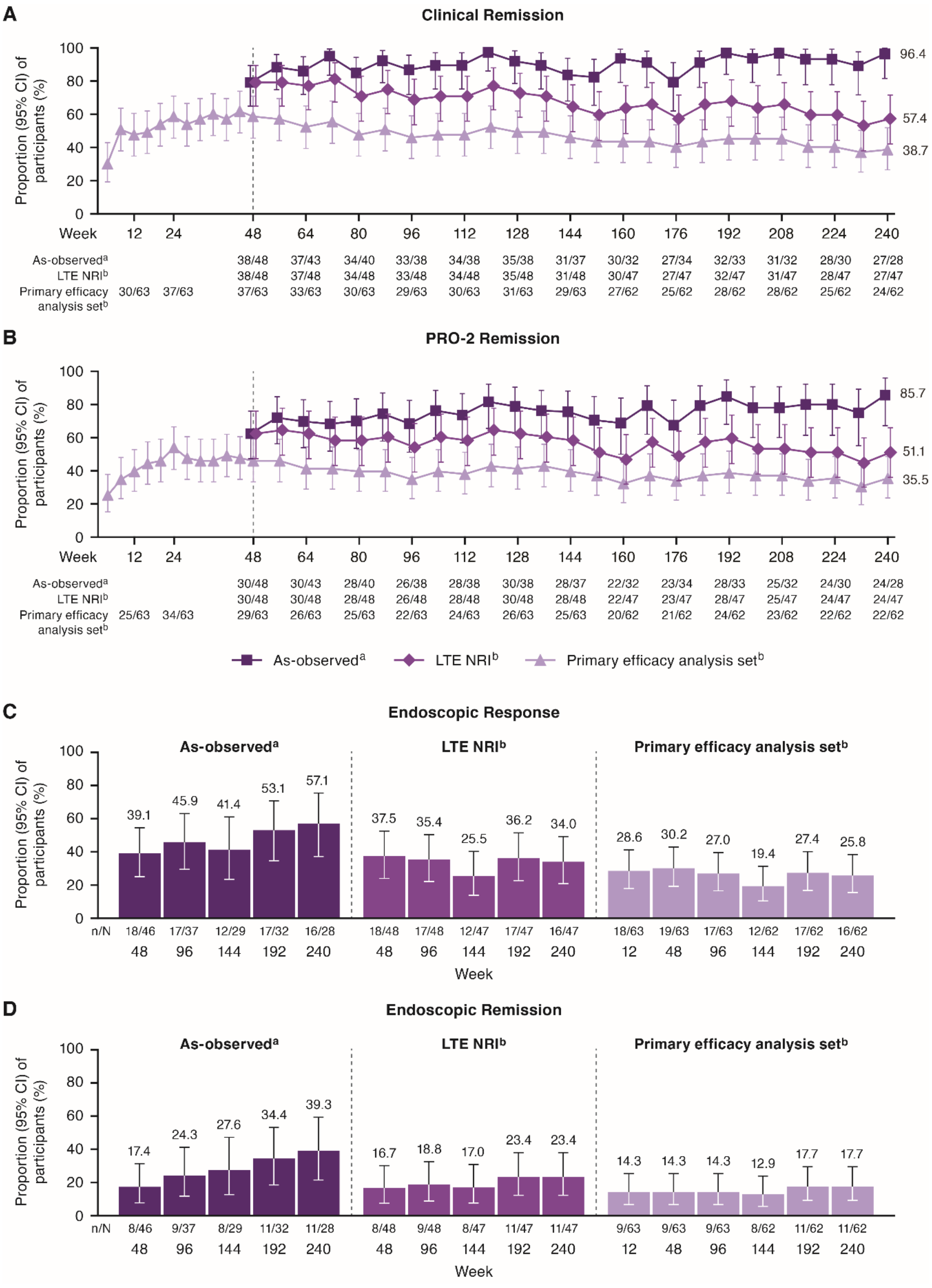

[a] Observed rates were evaluated in participants who entered the LTE and remained on treatment at that specific timepoint. Participants who dose-adjusted were excluded after the time of dose adjustment. No treatment failure rules were applied. Participants who did not have data at a visit were excluded from analysis for that visit.
[b] Participants with missing data at a particular timepoint after accounting for intercurrent event strategies were considered not to have achieved the endpoint at that timepoint.
Participants who completed the study at Week 144 under a previous version of the protocol are excluded from the denominator for analyses at timepoints after Week 144. These participants were not scheduled to have an ileocolonoscopy at Week 144 and were excluded from analysis of endoscopic response and endoscopic remission at Week 144 and thereafter.
Clinical remission is defined as CDAI score <150. PRO-2 remission is defined as AP mean daily score ≤1 and SF mean daily score ≤3, and no worsening of AP or SF from baseline. Endoscopic response is defined as ≥50% improvement from baseline in SES-CD or SES-CD ≤2. Endoscopic remission is defined as SES-CD ≤4 with at least a 2-point reduction from baseline and no subscore >1 in any subcomponent.
AP=Abdominal pain; CDAI=Crohn's Disease Activity Index; CI=Confidence interval; LTE=Long-term extension; NRI=Non-responder imputation; PRO=Patient-reported outcome; SES-CD=Simple Endoscopic Score for Crohn's Disease; SF=Stool frequency.

Supplementary Figure 8. Median serum pre-administration guselkumab concentrations (µg/mL) from Week 48 through Week 240

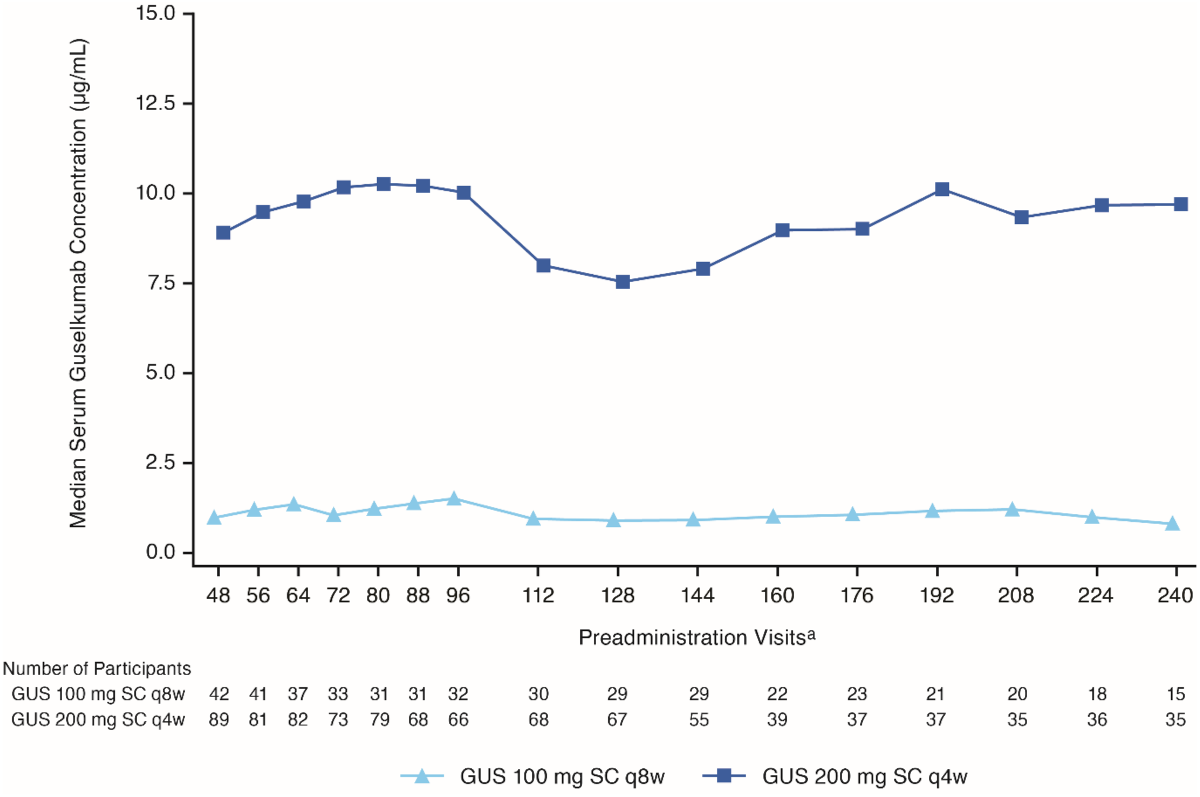

[a] Visits included are those at which both guselkumab dosing and sample collection occurred.
For participants who were dose-adjusted to guselkumab 200 mg SC, concentrations after the dose adjustment were not included in the analysis.
The guselkumab LTE PK analysis set included randomized participants who entered the LTE phase of the study, received ≥1 dose of guselkumab and had ≥1 valid blood sample drawn for PK analysis during the LTE.
GUS=guselkumab; LTE=Long-term extension; PK=Pharmacokinetics; q4w=Every 4 weeks; q8w=Every 8 weeks; SC=Subcutaneous.

Supplementary Figure 9. Proportions of LTE participants who were in (A) clinical remission and (B) endoscopic response at Week 240 in the combined guselkumab group by guselkumab concentration quartiles (LTE PK analysis set)

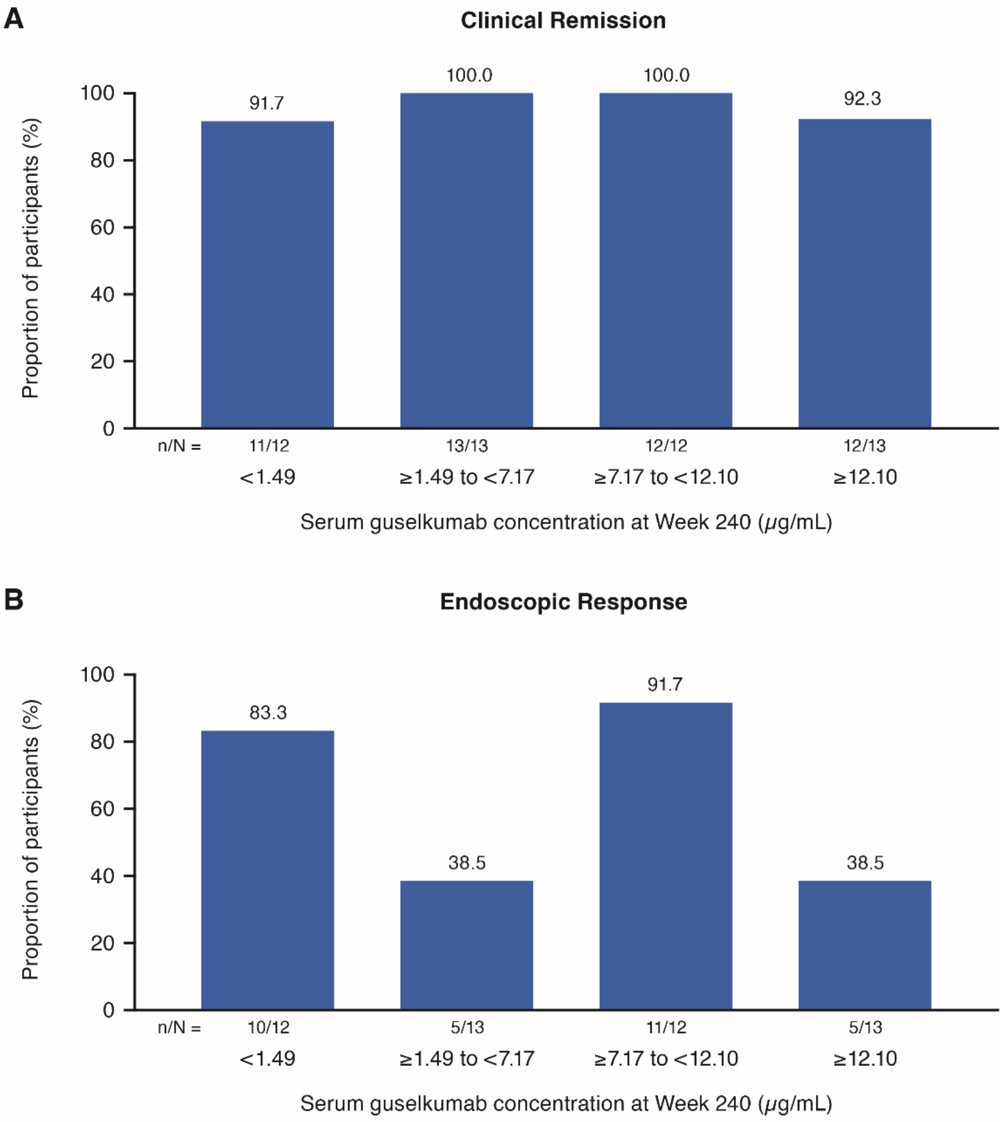

The guselkumab LTE PK analysis set is defined as randomized participants who entered the LTE phase of the study, have received ≥1 dose of guselkumab and have ≥1 valid blood sample drawn for PK analysis during the LTE. Participants who had a dose adjustment (actual or sham) were not included in the analysis.
Handling of missing data: Participants who had a Crohn's disease-related surgery or discontinued study agent due to lack of efficacy or an AE of worsening Crohn's disease prior to Week 240 were considered not to be in clinical remission at Week 240.
Participants who had discontinued study agent due to reasons other than COVID-19 restrictions/issues, lack of efficacy or AE of worsening Crohn's disease at Week 240 had their observed Week 240 data used, if available, to determine responder and non-responder status at Week 240. Participants who had discontinued study agent due to COVID-19 restrictions/issues prior to Week 240 did not have their data used at Week 240. After applying these rules, participants with missing data at Week 240 were considered not to have achieved the endpoint at Week 240.

Clinical remission is defined as CDAI score <150. Endoscopic response is defined as at least 50% improvement from baseline in SES-CD or SES-CD ≤2.
AE=Adverse event; CDAI=Crohn's Disease Activity Index; COVID-19=Coronavirus disease 2019; LTE=Long-term extension; PK=Pharmacokinetics; SES-CD=Simple Endoscopic Score for Crohn's Disease.
